# *Legionella* effectors SidC/SdcA ubiquitinate multiple small GTPases and SNARE proteins to promote phagosomal maturation

**DOI:** 10.1007/s00018-024-05271-7

**Published:** 2024-06-05

**Authors:** Kelong Ma, Rundong Shu, Hongtao Liu, Jinli Ge, Jiayang Liu, Qian Lu, Jiaqi Fu, Xiaoyun Liu, Jiazhang Qiu

**Affiliations:** 1 State Key Laboratory for Diagnosis and Treatment of Severe Zoonotic Infectious Diseases, Key Laboratory for Zoonosis Research of the Ministry of Education, College of Veterinary Medicine, Jilin University, Center for Pathogen Biology and Infectious Diseases, The First Hospital of Jilin University, Changchun, China; 2 Department of Microbiology and Infectious Disease Center, School of Basic Medical Sciences, Peking University Health Science Center, Beijing, China; 3 NHC Key Laboratory of Medical Immunology, Peking University, Beijing, China

**Keywords:** Legionella pneumophila, Effector proteins, Rab small GTPase, SNARE proteins, Ubiquitination, RILP

## Abstract

Protein ubiquitination is one of the most important posttranslational modifications (PTMs) in eukaryotes and is involved in the regulation of almost all cellular signaling pathways. The intracellular bacterial pathogen *Legionella pneumophila* translocates at least 26 effectors to hijack host ubiquitination signaling via distinct mechanisms. Among these effectors, SidC/SdcA are novel E3 ubiquitin ligases with the adoption of a Cys-His-Asp catalytic triad. SidC/SdcA are critical for the recruitment of endoplasmic reticulum (ER)-derived vesicles to the *Legionella*-*c*ontaining *v*acuole (LCV). However, the ubiquitination targets of SidC/SdcA are largely unknown, which restricts our understanding of the mechanisms used by these effectors to hijack the vesicle trafficking pathway. Here, we demonstrated that multiple Rab small GTPases and target soluble N-ethylmaleimide-sensitive factor attachment protein receptors (SNARE) proteins are *bona fide* ubiquitination substrates of SidC/SdcA. SidC/SdcA-mediated ubiquitination of syntaxin 3 and syntaxin 4 promotes their unconventional pairing with the vesicle-SNARE protein Sec22b, thereby contributing to the membrane fusion of ER-derived vesicles with the phagosome. In addition, our data reveal that ubiquitination of Rab7 by SidC/SdcA is critical for its association with the LCV membrane. Rab7 ubiquitination could impair its binding with the downstream effector Rab-interacting lysosomal protein (RILP), which partially explains why LCVs avoid fusion with lysosomes despite the acquisition of Rab7. Taken together, our study reveals the biological mechanisms employed by SidC/SdcA to promote the maturation of the LCVs.

**Supplementary Information:**

The online version contains supplementary material available at 10.1007/s00018-024-05271-7.

## Introduction

*Legionella pneumophila* is a gram-negative facultative intracellular bacterium ubiquitously found in natural environments where it parasitizes free-living amoebae [1]. Upon inhalation of bacteria-ridden aerosols, *L. pneumophila* is able to survive and replicate in lung macrophages, thus causing life-threatening Legionnaires’ disease, a severe type of pneumonia [2]. After being taken up by host cells, the bacterium resides in a membrane-enclosed compartment termed the *Legionella*-containing vacuole (LCV), which avoids fusion with lysosomes and supports intracellular *L. pneumophila* proliferation [3]. The biogenesis and maturation of LCVs is strictly dependent on the *d*efect in *o*rganelle *t*rafficking (Dot)/*i*ntra*c*ellular *m*ultiplication (Icm) type IV secretion system that injects a large cohort of effector proteins (over 330) into the host cytosol [4]. Once translocated, effector proteins target specific host substrates to alter or hijack diverse host signaling pathways, thereby facilitating the remodeling of LCVs or compromising host antimicrobial mechanisms [4]. Therefore, studies on the biochemical functions and biological relevance of these effectors are of critical importance to further our understanding on *L. pneumophila* pathogenesis. For instance, the small GTPase Rab1, one of the critical regulatory factors involved in endoplasmic reticulum (ER)-to-Golgi vesicle trafficking, is extensively targeted by multiple Dot/Icm substrates, including SidM/DrrA [5–7], SidD [8, 9], LepB [10], AnkX [11, 12], Lem3 [12] and SidEs [13], thus promoting the recruitment and fusion of ER-derived vesicles to the LCV membrane; the effector protein MavQ functions coordinately with LepB and SidF to *de novo* biosynthesize phosphatidylinositol 4-phosphate (PtdIns4P), one of the hallmarks of the mature LCV membrane [14].

One of the most frequent and efficient strategies used by bacterial effector proteins for the interference of host functions is to catalyze posttranslational modifications (PTMs) of specific host factors [15], such as phosphorylation [16], ubiquitination [17], methylation [18–20], ADP-ribosylation [21, 22], and glucosylation [23]. Among these PTMs, ubiquitination has been demonstrated to regulate almost every eukaryotic biological process, including innate and adaptive immunity [24]. Ubiquitination is accomplished through the sequential actions of E1 ubiquitin-activating enzyme, E2 ubiquitin-conjugating enzyme, and E3 ubiquitin ligase [25, 26]. Ubiquitin is first activated by the E1-activating enzyme at the expense of adenosine triphosphate (ATP) and forms thioester-linkage between its carboxyl-terminus and an active site cysteine of E1. Then, the E1-loaded ubiquitin is transferred to the cysteine residue on E2-conjugating enzymes by a transthiolation reaction. Finally, E3-ligases catalyze the covalent attachment of ubiquitin to the substrate proteins, usually via isopeptide bonds formed by the ubiquitin carboxyl-terminal glycine residue and the substrate lysine residues [25]. Ubiquitination is divided into monoubiquitination, multiubiquitination, or polyubiquitination, which result in either proteasomal degradation or signal transduction [25]. Akin to most PTMs, ubiquitination is a reversible process that is performed by deubiquitinases (DUBs), which remove ubiquitin modifications from substrates [27]. Despite the absence of a ubiquitination system, many intracellular pathogens, including bacteria and viruses, have evolved strategies to hijack ubiquitin signaling in their hosts [28]. One such mechanism utilized by intracellular bacteria is encoding effector proteins that mimic E3 ligase or DUB activity [17]. For example, the *Yersinia* Type III secretion system effector YopM functions as an E3 ubiquitin ligase that targets NLRP3 to induce necrotic cell death [29]. *Coxiella burnetii* type IV secretion effector EmcB is identified as a DUB that suppresses RIG-I signaling, thereby antagonizing host immune surveillance [30].

*L. pneumophila* encodes at least 26 Dot/Icm effectors that function to modulate host ubiquitination signaling. Biochemical and structural studies have revealed that some of them adopt classic E3 ligase or DUB domains, whereas others have evolved novel catalytic folds or even noncanonical biochemical reactions [31]. For instance, LegU2/LubX [32, 33], GobX [33], and RavN [34] are *L. pneumophila* E3 ligases that harbor conserved U-box domains; SidC and its paralog SdcA exhibit E3 ligase activity by virtue of the presence of a Cys-His-Asp catalytic triad [35], a motif usually identified in cysteine proteases and DUBs. Strikingly, SidE family effector proteins (SidEs) catalyze an unconventional, ATP/E1/E2-independent, and NAD-dependent ubiquitination reaction by the sequential actions of mono-ADP-ribosyltransferase (mART) and phosphodiesterase (PDE) activities [13, 36, 37]. SidE-mediated ubiquitination involves the conjugation of ubiquitin molecules to substrate serine residues via a phosphoribosyl (PR) linkage [36]. PR-linked serine ubiquitination is specifically reversed by the effectors DupA/DupB [38, 39], which possess PDE domains. However, the host substrates and biological functions of most of these ubiquitination-modulating effectors are largely unknown, which greatly restricted our understanding on their roles in the virulence of *L. pneumophila*.

To construct a suitable environment for replication, LCVs intimately communicate with host secretory and retrograde vesicle trafficking pathways and eventually develop into a compartment resembling the ER [3, 40]. Importantly, although LCVs avoid fusion with lysosomes, they still communicate with the endosomal pathway, as evidenced by the association of the late endosomal small GTPase Rab7 on LCVs [41, 42]. In the present study, we found that multiple Rab small GTPases, including Rab7, are ubiquitination substrates of the *L. pneumophila* effector proteins SidC and SdcA. SidC/SdcA is critical for the association of Rab7 with the LCV membrane in a manner dependent on its E3 ligase activity. Importantly, when Rab7 undergoes ubiquitination, the binding ability between Rab7 and its downstream effector Rab-interacting lysosomal protein (RILP) is impaired, which partially elucidates the mechanism used by *L. pneumophila* to escape phagosome-lysosome fusion despite the acquisition of Rab7 on LCVs. In addition, we also found that multiple target soluble N-ethylmaleimide-sensitive factor attachment protein receptors (t-SNARE), including syntaxin (STX) 3 and 4, are also targeted by SidC/SdcA for ubiquitination in *L. pneumophila* infection, which could increase its noncanonical pairing with the vesicle SNARE (v-SNARE) protein Sec22b. Together, these findings reveal the mechanistic insights of SidC/SdcA to hijack host vesicle trafficking pathways and evade lysosomal fusion.

## Results

### The E3 ligase activity of SidC/SdcA is required for optimal growth of *L. Pneumophila*

**Fig. 1:**
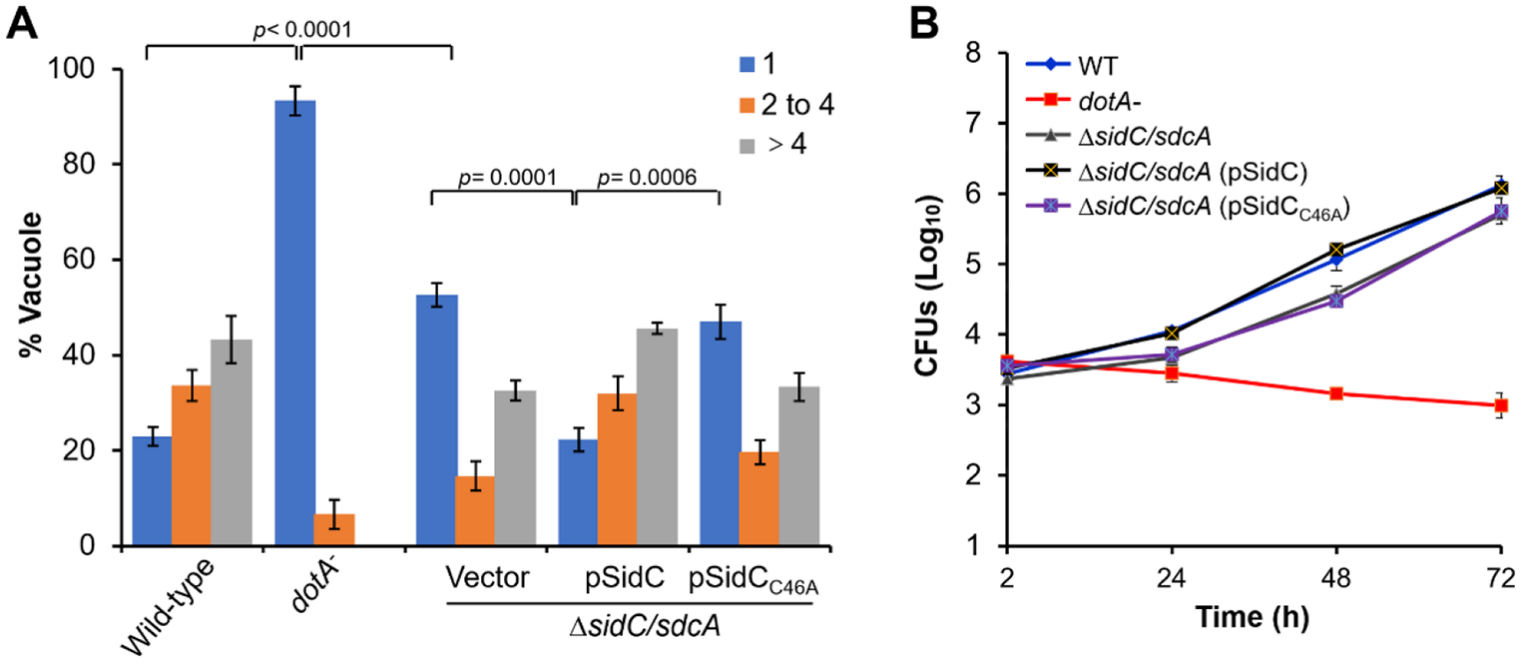
The E3 ubiquitin ligase activity of SidC/SdcA is required for optimal intracellular *L. pneumophila* growth. Bone marrow-derived macrophages (BMDMs) were infected with wild-type, *dotA*^−^, Δ*sidC/sdcA*, Δ*sidC/sdcA* (pSidC), and Δ*sidC/sdcA* (pSidC_C46A_) *L. pneumophila* strains at an MOI of 0.05. (**A**) Infected cells were fixed at 8 h postinfection and subjected to immunostaining with anti-*Legionella* antibodies. The number of bacteria residing in the vacuole was scored under a fluorescence microscope. One hundred phagosomes were calculated for each infection sample. (**B**) Intracellular growth of the bacterial strains was determined at each examined time point by counting the colony forming units (CFUs) on the plates. Data in panel A are presented as the mean ± standard deviation (SD) of three independent tests, and panel B is one representative from three independent experiments performed in triplicate

SidC and its paralog protein SdcA are *L. pneumophila* Dot/Icm substrates with established biochemical functions. The C-terminal 20 kDa fragment of SidC/SdcA comprises a phosphotidylinositiol 4-phosphate (PI4P) binding domain, which is required for its anchoring to the phagosomal membrane [43]. The N-terminal portion of SidC/SdcA possesses unique E3 ligase activity [35]. SidC/SdcA has been shown to be required for the optimal growth of *L. pneumophila* within bone marrow-derived macrophages (BMDMs) [44]. To further determine whether the E3 ligase activity is responsible for the growth phenotypes of SidC/SdcA, we infected BMDMs with wild-type, *dotA*^−^, Δ*sidC/sdcA*, Δ*sidC/sdcA* (pSidC), and Δ*sidC/sdcA* (pSidC_C46A_) *L. pneumophila* strains for indicated durations. Consistent with previous observations [44], we found that the Δ*sidC/sdcA* mutant strain formed a significantly higher percentage of nonreplicating vacuoles than the cells infected with wild-type *L. pneumophila* at 8 h post infection of BMDMs (Fig. 1A). In wild-type infection, the percentages of vacuoles harboring a single bacterium, two to four bacteria, and more than 4 bacteria were 23%, 33.7%, and 43.3% (Fig. 1A), respectively. In contrast, infection of cells with the Δ*sidC/sdcA* strain resulted in 52.7% of vacuoles containing a single bacterium, 14.7% of vacuoles containing two to four bacteria, and 32.6% of vacuoles containing more than four bacteria (Fig. 1A). Importantly, the defect in the formation of replicating vacuoles can be restored by the expression of wild-type SidC but not the E3 ligase-deficient SidC mutant (SidC_C46A_) from a plasmid in the Δ*sidC/sdcA* mutant (Fig. 1A). Apparently, the mutation in C46A did not cause defects in the expression and translocation of SidC (Figure S1). Moreover, an intracellular growth curve determination showed that the absence of *sidC/sdcA* led to a modest growth defect in BMDMs, and this defect could be fully complemented by wild-type but not by C46A mutant SidC (Fig. 1B). Taken together, our data indicate that the E3 ligase activity of SidC/SdcA is required for optimal intracellular *L. pneumophila* replication.

### Ubiquitinome analysis of *L. pneumophila*-infected cells identified multiple small GTPase and t-SNARE proteins that are targeted by SidC/SdcA

**Fig. 2:**
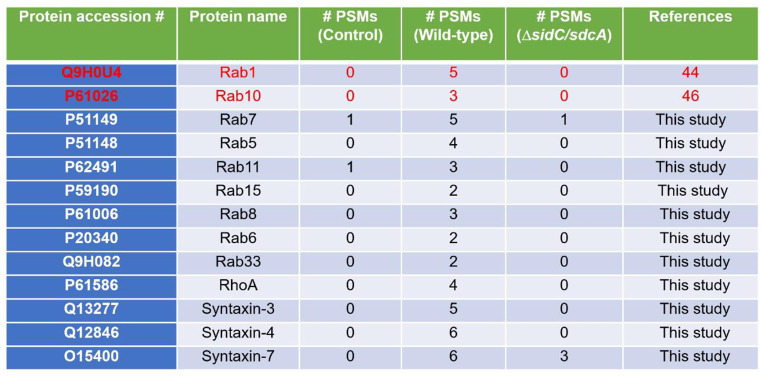
Candidate ubiquitination substrates of SidC/SdcA identified from *L. pneumophila*-infected cells by liquid chromatography-tandem mass spectrometry (LC‒MS/MS). HEK293 cells transfected with 3xHA-Ub and FcγII were either left uninfected or challenged with the wild-type or Δ*sidC/sdcA L. pneumophila* strain at an MOI of 20 for 2 h. The anti-HA immunoprecipitated products of the cell lysates were digested by trypsin and further analyzed by LC‒MS/MS

SidC and SdcA were first identified as tethering factors for the recruitment of ER-derived vesicles to the LCV membrane, thus promoting maturation of the LCVs [43]. Our previous study revealed that the E3 ligase activity of SidC/SdcA is essential for the recruitment of the ER marker GFP-HDEL to the bacterial phagosome [35]. Therefore, it is reasonable to infer that SidC might target specific host proteins for ubiquitination, thereby redirecting vesicle trafficking routes. However, the ubiquitination substrates of SidC/SdcA remained largely unknown. To this end, we performed a ubiquitinome analysis of 3xHA-ubiquitin-expressing cells that were infected with wild-type or Δ*sidC/sdcA* mutant *L. pneumophila* strains [45]. After mass spectrometry (MS) analysis, we obtained a list of potential ubiquitination targets of SidC/SdcA [45]. Notably, Rab1 and Rab10, two previously established substrates of SidC/SdcA [44, 46], were identified in this attempt (Fig. 2), supporting the reliability of this analysis.

In addition to the confirmed targets, multiple small GTPases in the Rho and Rab subfamilies, including RhoA, Rab5, Rab6, Rab7, Rab8, Rab11, Rab15, and Rab33, were differentially ubiquitinated between wild-type and Δ*sidC/sdcA L. pneumophila*-infected samples (Fig. 2). In addition, we also noticed that many t-SNARE proteins that participate in the regulation of vesicle trafficking, such as STX3, STX4, and STX7, were present in the list of candidates (Fig. 2). Taken together, our ubiquitinome data indicate that SidC/SdcA potentially targets diverse regulators in the vesicle transport pathway.

### Validation of the ubiquitination substrates of SidC/SdcA

**Fig. 3:**
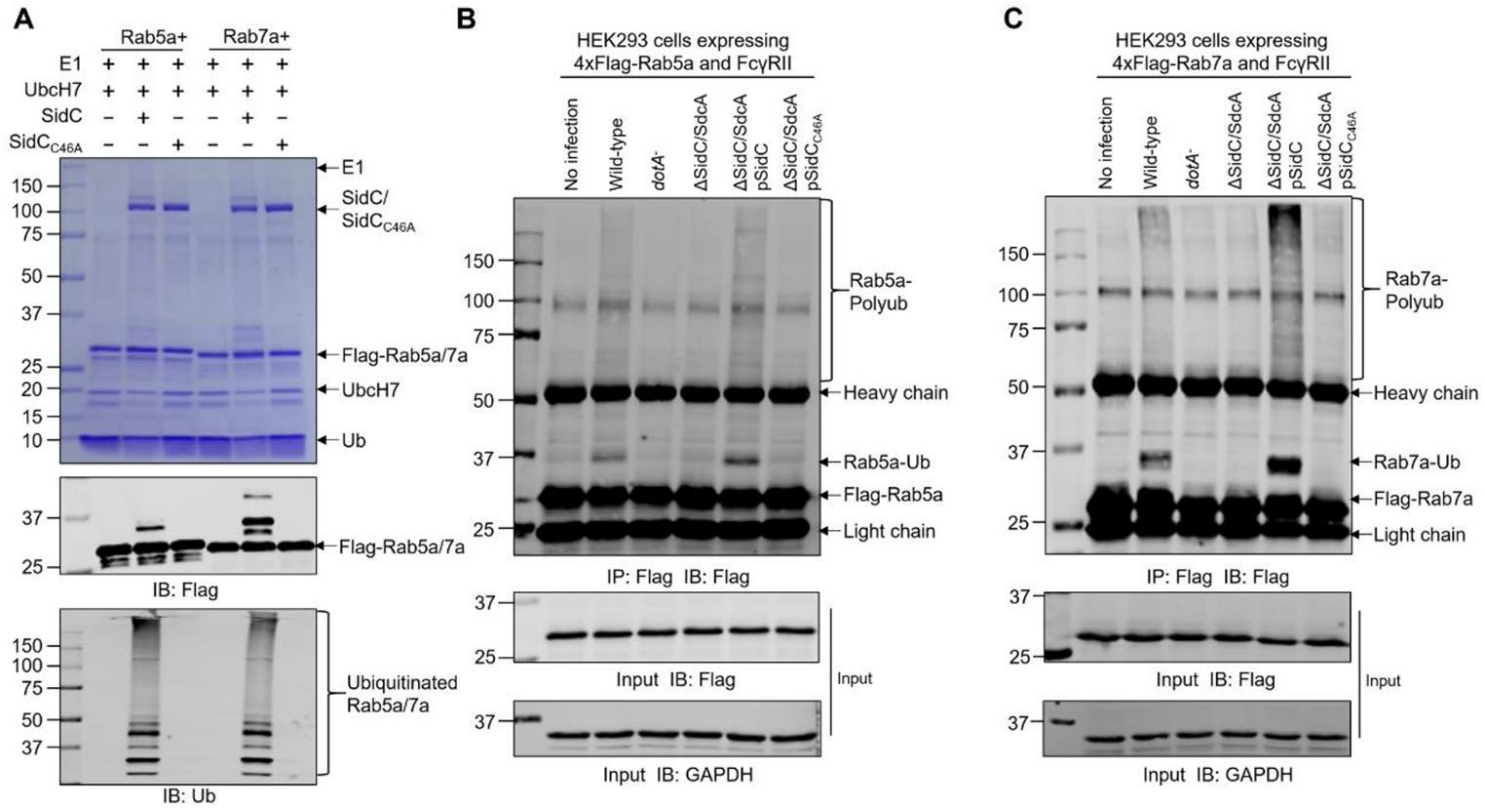
Ubiquitination of Rab5 and Rab7 by the effector protein SidC. (**A**) In vitro ubiquitination reactions containing E1, UbcH7, ubiquitin, 4xFlag-Rab5/Rab7, and SidC/SidC_C46A_ were performed at 37 °C for 1 h. After termination by the addition of SDS sampling buffer, protein samples were separated by SDS‒PAGE, and Rab5/Rab7 ubiquitination was visualized by Coomassie brilliant blue (CBB) staining (upper) or Western blot analysis with anti-Flag (middle) and anti-(ubiquitin) Ub (lower) antibodies. (**B-C**) HEK293 cells expressing 4xFlag-Rab5/Rab7 and FcγRII were infected with the indicated *L. pneumophila* strains (MOI = 50). Cell lysates were prepared at 2 h postinfection with RIPA buffer, and 4xFlag-Rab5 (B) and Rab7 (C) were enriched with anti-Flag agarose and detected by Western blot using anti-Flag antibodies. The anti-glyceraldehyde-3-phosphate dehydrogenase (GAPDH) blot was included to indicate equal loading. The data shown in A, B, and C are representative of three independent experiments

To validate the MS data, we first purified the recombinant small GTPase, SidC/SdcA and its catalytically inactive mutant. These proteins were further used in the in vitro ubiquitination assays. Ubiquitinated Rab5, Rab6, Rab7, Rab8, and Rab11 were observed in the ubiquitination reaction containing wild-type but not C46A mutant SidC (Fig. 3A and S2A). In addition, these small GTPases were also modified by SdcA in a catalytic cysteine-dependent manner (Figures S2B-C). As we failed to ectopically express full-length SidC in mammalian cells by transfection despite extensive attempts, we next coexpressed GFP-tagged SdcA with each of the small GTPases fused with an N-terminal 4xFlag tag in HEK293T cells. After immunoprecipitation, except for Rab8, modified forms of the in vitro confirmed substrates were detected in cells producing GFP-SdcA but not GFP-SdcA_C45A_ (Figure S3). Taken together, these in vitro and in vivo data validate that multiple Rab small GTPases implicated in the secretory or endocytic trafficking pathways are targeted by SidC and SdcA.

Next, we investigated the modification of the abovementioned substrates under infection conditions. To this end, we first transfected HEK293 cells with each of the 4xFlag-tagged target proteins followed by infection with wild-type, *dotA*^−^, Δ*sidC/sdcA*, Δ*sidC/sdcA* (pSidC), and Δ*sidC/sdcA* (pSidC_C46A_) *L. pneumophila* strains. Western blot analysis of the anti-Flag immunoprecipitates revealed that Rab small GTPases, including Rab5, Rab6, Rab7, Rab8, and Rab11, were ubiquitinated by SidC/SdcA in an active cysteine-dependent fashion (Fig. 3B-C and S4A-C), establishing that these proteins are physiological substrates of SidC/SdcA.

**Fig. 4:**
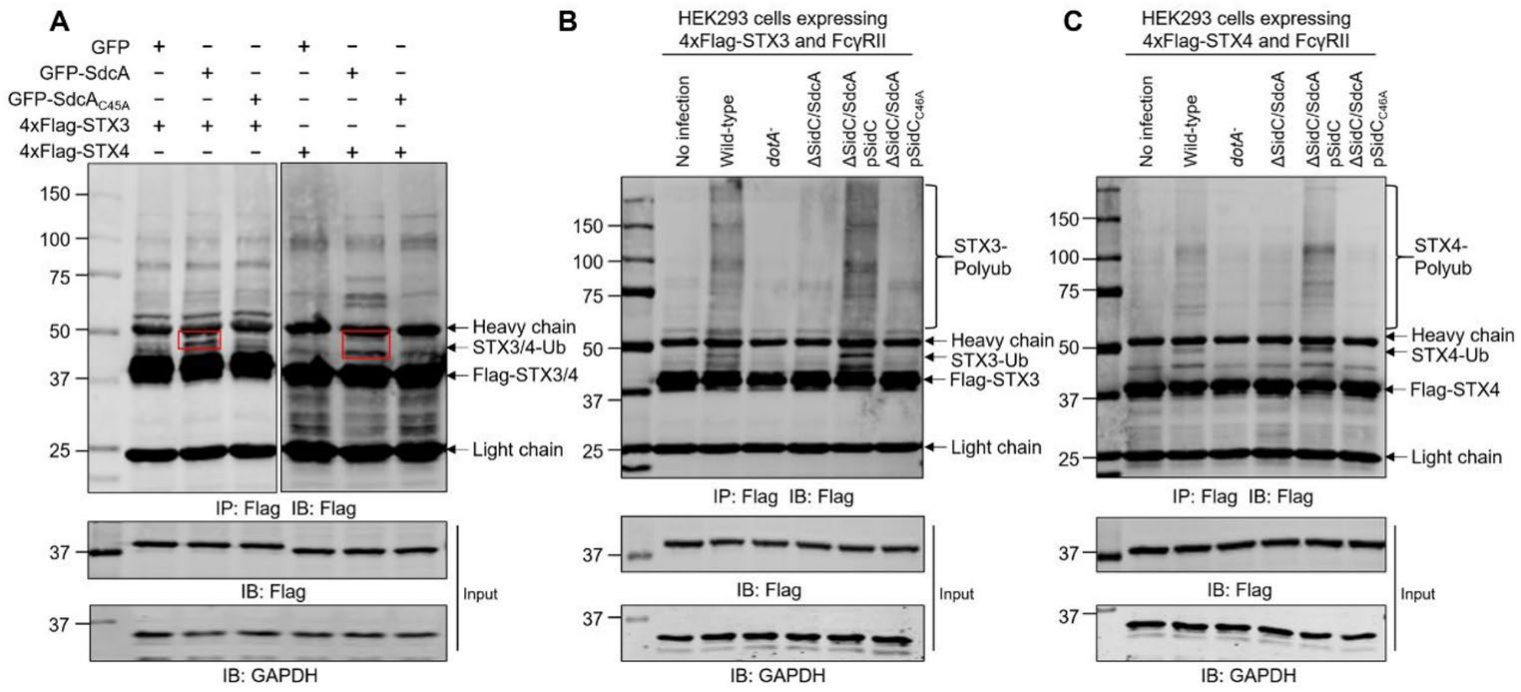
SidC/SdcA induced ubiquitination of the t-SNARE proteins STX3 and STX4. (**A**) 4xFlag-STX3/STX4 was cotransfected with GFP, GFP-SdcA, or GFP-SdcA_C45A_ into HEK293T cells. Cell lysates prepared at 24 h posttransfection were immunoprecipitated by anti-Flag agarose and analyzed by Western blot with an anti-Flag antibody. The red circle shows the modified forms of syntaxin 3 (STX3) and syntaxin 4 (STX4). (**B-C**) HEK293 cells transfected to produce 4xFlag-STX3/STX4 and FcγRII were either uninfected (lane 1) or infected with wild-type, *dotA*^−^, Δ*sidC/sdcA*, Δ*sidC/sdcA* (pSidC), and Δ*sidC/sdcA* (pSidC_C46A_) *L. pneumophila* strains (lanes 2–6). At 2 h after infection, the cells were lysed, immunoprecipitated and probed with the anti-Flag antibody. The lysates probed with the antibody specific for anti-glyceraldehyde-3-phosphate dehydrogenase (GAPDH) were used as a loading control. The data shown are one representative of three independent experiments

Since we were unable to purify soluble recombinant STXs from *E. coli*, we coexpressed GFP-SdcA with 4xFlag-STX3/4/7 to validate the modification imposed by the E3 ligase activity of SidC/SdcA. Following immunoprecipitation, ubiquitination of STX3, STX4 and STX7 occurred in cells cotransfected with wild-type SdcA but not its C45A mutant (Fig. 4A and S5A). Moreover, ubiquitination of these t-SNARE proteins was detected in cells infected with wild-type but not Δ*sidC/sdcA L. pneumophila* (Fig. 4B-C and S5B). Importantly, modification of these STXs was complemented by introducing a plasmid carrying wild-type SidC but not its C46A mutant. Therefore, in addition to those small GTPases, t-SNARE proteins including STX3, STX4, and STX7 are also ubiquitination substrates of SidC/SdcA.

### Ubiquitination of STX3 and STX4 by SidC/SdcA promotes its noncanonical pairing with Sec22b

**Fig. 5:**
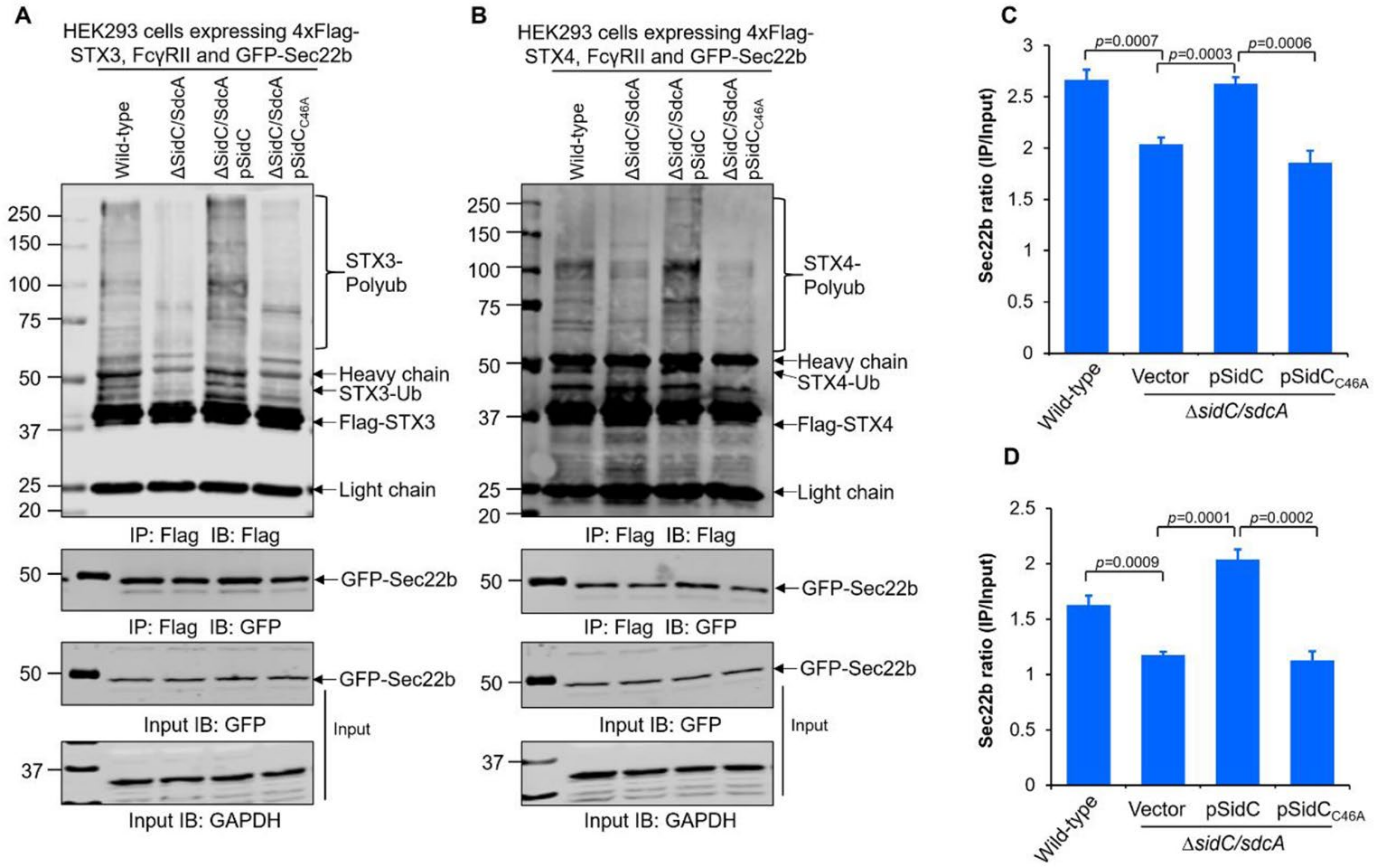
Ubiquitination of syntaxin 3 (STX3) and syntaxin 4 (STX4) promotes their noncanonical pairing with the v-SNARE protein Sec22b during *L. pneumophila* infection. (**A-B**) Plasmids encoding 4xFlag-STX3/STX4, GFP-Sec22b, and FcγRII were transfected into HEK293 cells. At 24 h posttransfection, cells were then challenged with relevant *L. pneumophila* strains (lanes 2–5) for 2 h (MOI = 50). 4xFlag-STX3/STX4 was enriched by immunoprecipitation of the cell lysates with anti-Flag agarose, and the bead-bound proteins were further detected by Western blotting with anti-Flag and anti-GFP antibodies. The cell lysates probed with the anti-glyceraldehyde-3-phosphate dehydrogenase (GAPDH) antibody were included as a loading control. (**C-D**) Intensities of the GFP-Sec22b bands were quantified by ImageJ software. Noncanonical pairing between STX3 (C, related to panel A), STX4 (D, related to panel B) and Sec22b was evaluated by calculating the GFP-Sec22b ratio (IP/Input). The results shown in panels A and B are representative of three independent experiments. Values in panels C and D are the mean ± SD of three independent tests

Previous studies have established that *L. pneumophila* infection causes unconventional interactions between plasma membrane t-SNAREs (e.g., STX3 and STX4) and v-SNARE Sec22b, contributing to LCV biogenesis [47]. In addition, it has been reported that deubiquitination of Sec22b by LotB, an ovarian tumor-related proteases (OTU) family DUB, stimulates the dissociation of STX3 from Sec22b [48], suggesting that Sec22b ubiquitination is required for such an interaction. Indeed, our recent study revealed that Lug15, a novel *L. pneumophila* E3 ligase, is responsible for Sec22b ubiquitination during infection and promotes the noncanonical interaction between STX3 and Sec22b [49]. Owing to the SidC/SdcA-catalyzed ubiquitination of STX3 and STX4, we hypothesized that SidC/SdcA may also play an important role in the noncanonical SNARE pairing. To test this hypothesis, we infected 4xFlag-tagged-STX3/STX4- and GFP-Sec22b-expressing HEK293 cells with the indicated *L. pneumophila* strains. The interaction between these proteins was evaluated by Western blot analysis of the anti-Flag immunoprecipitates. Indeed, cells challenged with the Δ*sidC/sdcA* mutant led to significantly lower levels of STX3- or STX4-associated Sec22b than those infected with the wild-type strain (Fig. 5A-D). This defect can be complemented by the plasmid-driven expression of wild-type but not C46A mutant SidC (Fig. 5A-D). Hence, these data indicate that the E3 ligase activity of SidC/SdcA participates in the fusion of ER-derived vesicles with the phagosome membrane that originates from the plasma membrane.

### SidC/SdcA ubiquitinates Rab7 at multiple lysine residues

**Fig. 6:**
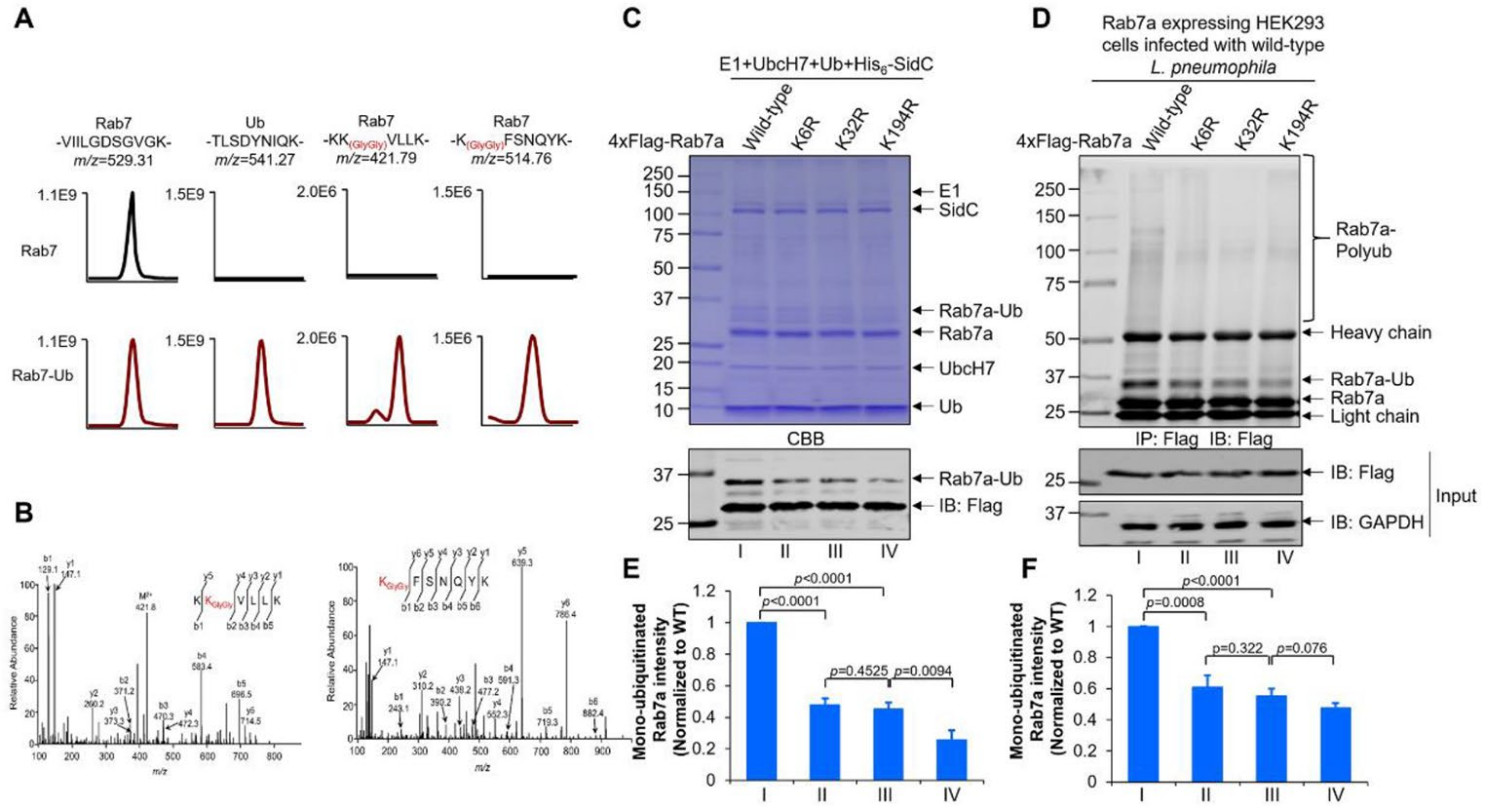
SidC/SdcA ubiquitinates Rab7 at multiple lysine residues. (**A**) Extracted ion chromatograms of the tryptic peptides (-KKVLLK-, and -KFSNQYK-) bearing di-glycine remnants at K6 and K32 of Rab7 are shown. The unmodified reference peptide of Rab7 (-VIILGDSGVGK-) and the ubiquitin peptide (-TLSDYNIQK-) are also presented. (**B**) Collision-induced dissociation (CID) MS/MS spectra of Rab7 peptides carrying di-glycine remnants at K6 and K32 are shown. (**C**) In vitro ubiquitination of Rab7 lysine mutants by SidC. Reactions consisting of E1, UbcH7, ubiquitin, His_6_-SidC and each of the Rab7 derivatives were incubated at 37 °C for 30 min. After termination of the reactions by supplementation with 5x SDS sample buffer, the proteins were resolved by SDS‒PAGE and visualized by Coomassie brilliant blue (CBB) staining or Western blot using the anti-Flag antibody. (**D**) HEK293 cells producing wild-type Rab7, Rab7_K6R_, Rab7_K32R_, or Rab7_K194R_ were infected with wild-type *L. pneumophila* for 2 h. Lysates of the infected cells were immunoprecipitated with anti-Flag beads and further analyzed by Western blotting with an antibody specific for Flag. Glyceraldehyde-3-phosphate dehydrogenase (GAPDH) was probed as the loading control. (**E-F**) Quantification of the mono-ubiquitinated Rab7a as shown in (C) and (D) was performed by ImageJ. Panel E is related to panel C while panel F is related to panel D. The data shown in panels C and D are representative of three independent assays. Values in panels E and F are the mean ± SD of three independent tests

To dissect the ubiquitination sites of Rab7 by SidC/SdcA, Rab7-Ub retrieved from the in vitro ubiquitination reaction was analyzed by MS analysis. We found that ubiquitin is covalently linked to the residues K6 and K32 in Rab7 (Fig. 6A-B). Signals of the modified peptides -KK_5(GlyGly)_VLLK- and -K_32(GlyGly)_FSNQYK- were detected in digested Rab7-Ub but not in the control samples (Fig. 6A-B). We next purified recombinant Rab7_K6R_ and Rab7_K32R_ and used them in an in vitro biochemical assay. Both mutants exhibited decreased ubiquitination by SidC compared to wild-type Rab7 (Fig. 6C and E). In addition, as Rab7 undergoes ubiquitination by mammalian E3 ligases at the K38, K191, and K194 residues [50], we also tested whether any of these sites are used by SidC. Interestingly, while Rab7_K38R_ and Rab7_K191R_ were modified by SidC as efficiently as the wild type, the formation of ubiquitinated species was markedly reduced in the reaction containing Rab7_K194R_ (Figure S6). Consistent with the in vitro observations, infection of Rab7_K6R−_, Rab7_K32R−_, and Rab7_K194R_-expressing cells with wild-type *L. pneumophila* strains yielded lower levels of ubiquitinated Rab7 (Fig. 6D and F). Taken together, these results indicate that Rab7 is modified by SidC/SdcA at multiple lysine residues.

### The E3 ligase activity of SidC/SdcA is critical for the association of Rab7 with the bacterial phagosomes

**Fig. 7:**
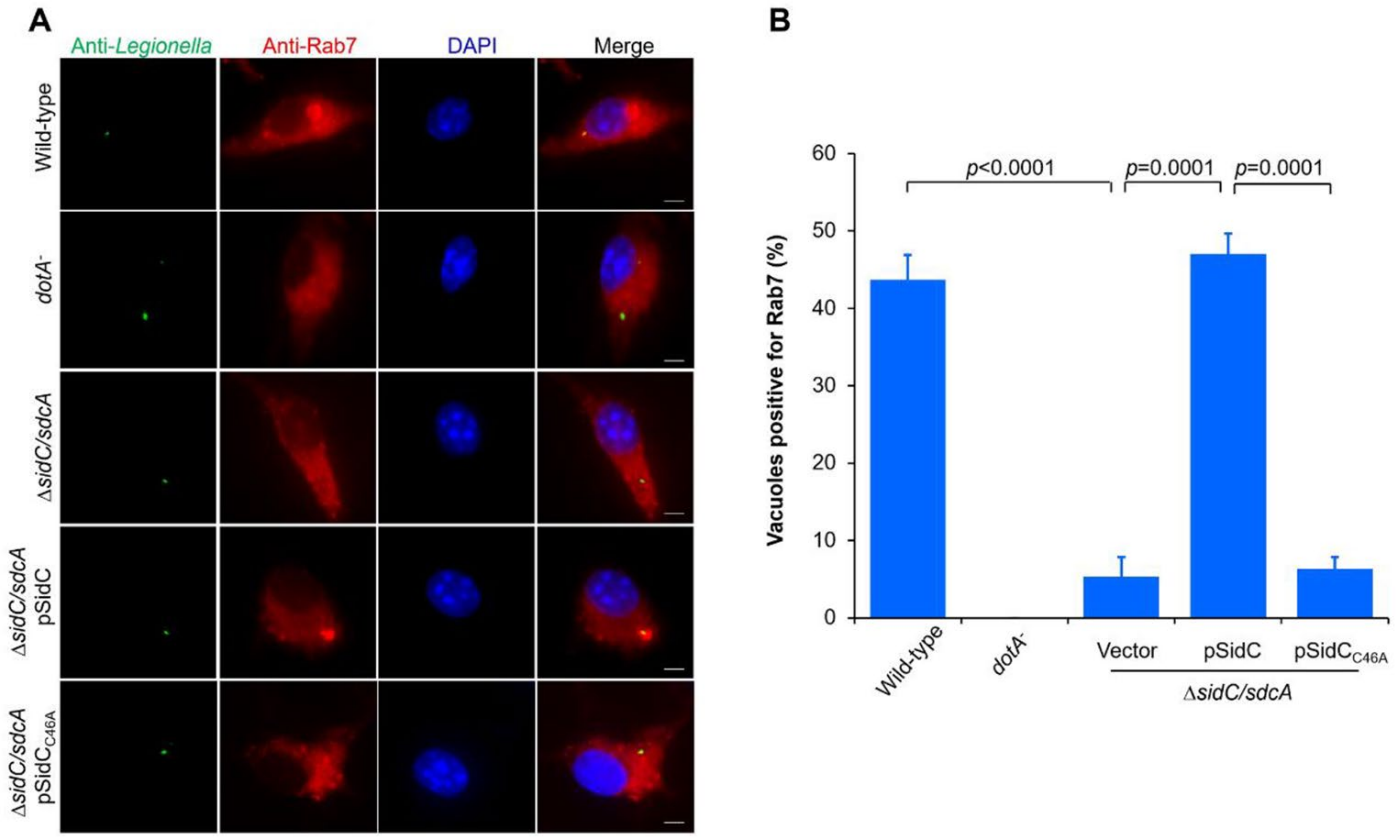
SidC/SdcA-dependent association of Rab7 with the bacterial phagosome. BMDMs were infected with wild-type, *dotA*^−^, Δ*sidC/sdcA*, Δ*sidC/sdcA* (pSidC), and Δ*sidC/sdcA* (pSidC_C46A_) *L. pneumophila* strains for 2 h at an MOI of 5. Fixed BMDMs were subjected to stepwise immunostaining with *Legionella*- (green) and Rab7- (red) specific antibodies. 2-(4-Amidinophenyl)-6-indolecarbamidine dihydrochloride (DAPI) staining was used to label the nucleus. Fluorescence signals were visualized under a Zeiss LSM880 confocal microscope. (**A**) Representative fluorescence micrographs of Rab7-positive and Rab7-negative LCVs in different *L. pneumophila* strain-challenged cells. Bar, 2 μm. (**B**) Percentage of Rab7-positive vacuoles. One hundred LCVs were scored for each infection sample, and values are the mean ± SD of three independent experiments

Rab7 is a late endosomal marker that promotes late steps of the endocytic pathway [51]. Rab7 was previously demonstrated to be recruited to the phagosomal membrane, suggesting that LCVs communicated not only with secretory but also endosomal pathways [41, 42]. Owing to the SidC/SdcA-mediated ubiquitination of Rab7, we next determined whether SidC/SdcA is required for the association of Rab7 with the LCVs. To this end, BMDMs were infected with relevant *L. pneumophila* strains and subjected to stepwise immunostaining with anti-*Legionella* and anti-Rab7 antibodies. Approximately 44% of the vacuoles containing wild-type bacteria were positively stained with Rab7 at 2 h post infection (Fig. 7A-B). In contrast, the *dotA*^−^ mutant *L. pneumophila* phagosomes failed to recruit Rab7 (Fig. 7A-B). Importantly, we found that only 5% of the LCVs containing Δ*sidC/sdcA* acquired Rab7. Complement of the mutant strain with a plasmid coding for wild-type but not C46A SidC could restore the percentage of Rab7-positive phagosomes to the level seen with wild-type *L. pneumophila* infection (Fig. 7A-B). Together, these observations indicate that SidC/SdcA is critical for Rab7 association with LCVs.

### SidC/SdcA contributes to the evasion of phagosome/lysosome fusion

**Fig. 8:**
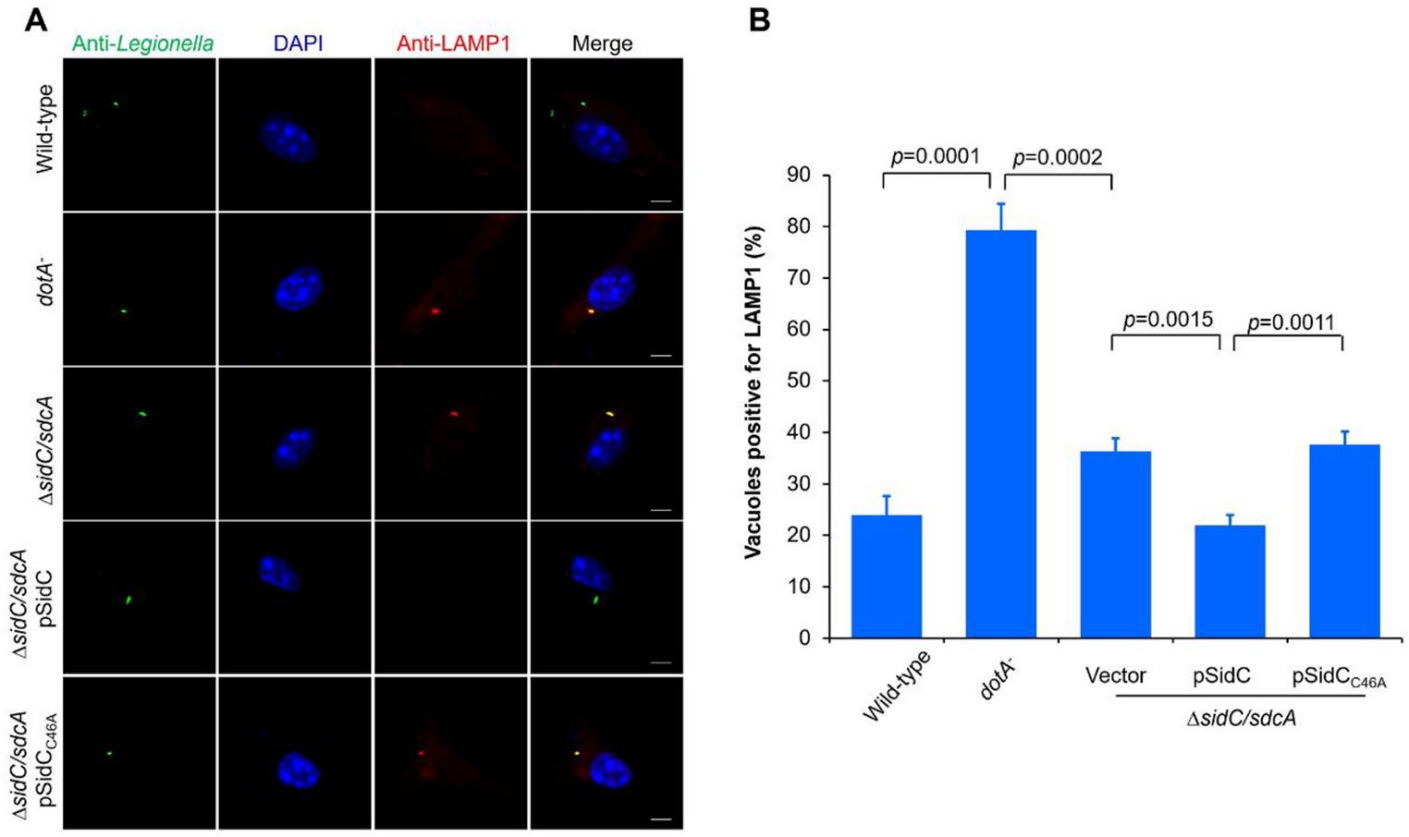
SidC/SdcA prevents the accumulation of LAMP1 on LCVs. BMDMs seeded on coverslips were challenged with the indicated *L. pneumophila* strains at an MOI of 5. Two hours after infection, the cells were fixed, permeabilized, and immunostained with anti-*Legionella* (green) and anti-lysosome-associated membrane proteins 1 (LAMP1) (red) antibodies. The nucleus was labeled by DAPI (blue). Immunofluorescence was observed using a Zeiss LSM880 confocal microscope. (**A**) Images show LAMP1 accumulation on LCVs harboring wild-type, *dotA*^−^, Δ*sidC/sdcA*, Δ*sidC/sdcA* (pSidC), and Δ*sidC/sdcA* (pSidC_C46A_) *L. pneumophila*. Bar, 2 μm. (**B**) Percentage of vacuoles positively stained with LAMP1. One hundred vacuoles were calculated for each sample. Data are presented as the mean ± SD from three independent assays

Rab7 is a key small GTPase that is implicated in late endosome/lysosome fusion [52]. Interestingly, despite the acquisition of Rab7, the LCVs avoid fusing with the lysosome, which is supported by the absence of lysosome-associated membrane proteins 1 (LAMP1) with the LCVs [53]. The abovementioned results have prompted us to hypothesize that SidC/SdcA may participate in the evasion of phagosomal fusion with lysosomes. To test this hypothesis, we infected BMDMs with relevant *L. pneumophila* strains and further immunostained the samples with antibodies specific for the bacteria and LAMP1. Wild-type strain infection caused only 24% LAMP1-associated vacuoles, while more than 79% of the phagosomes harboring the *dotA*^−^ mutant were positively stained with this lysosomal marker. Infection of the cells with the Δ*sidC/sdcA L. pneumophila* strain led to a significantly higher portion of LAMP1-positive LCVs (36%) than the wild-type infection (Fig. 8A-B). Moreover, the increase in LAMP1 association with the LCVs owing to the absence of SidC/SdcA could be restored to the level of wild-type strain-infected cells by plasmid-expressed wild-type SidC but not its catalytically inactive mutant SidC_C46A_ (Fig. 8A-B). Thus, these data establish the role of SidC/SdcA in preventing lysosomal fusion of LCVs.

### Ubiquitination of Rab7 by SidC/SdcA decreases its interaction with the downstream effector RILP

**Fig. 9:**
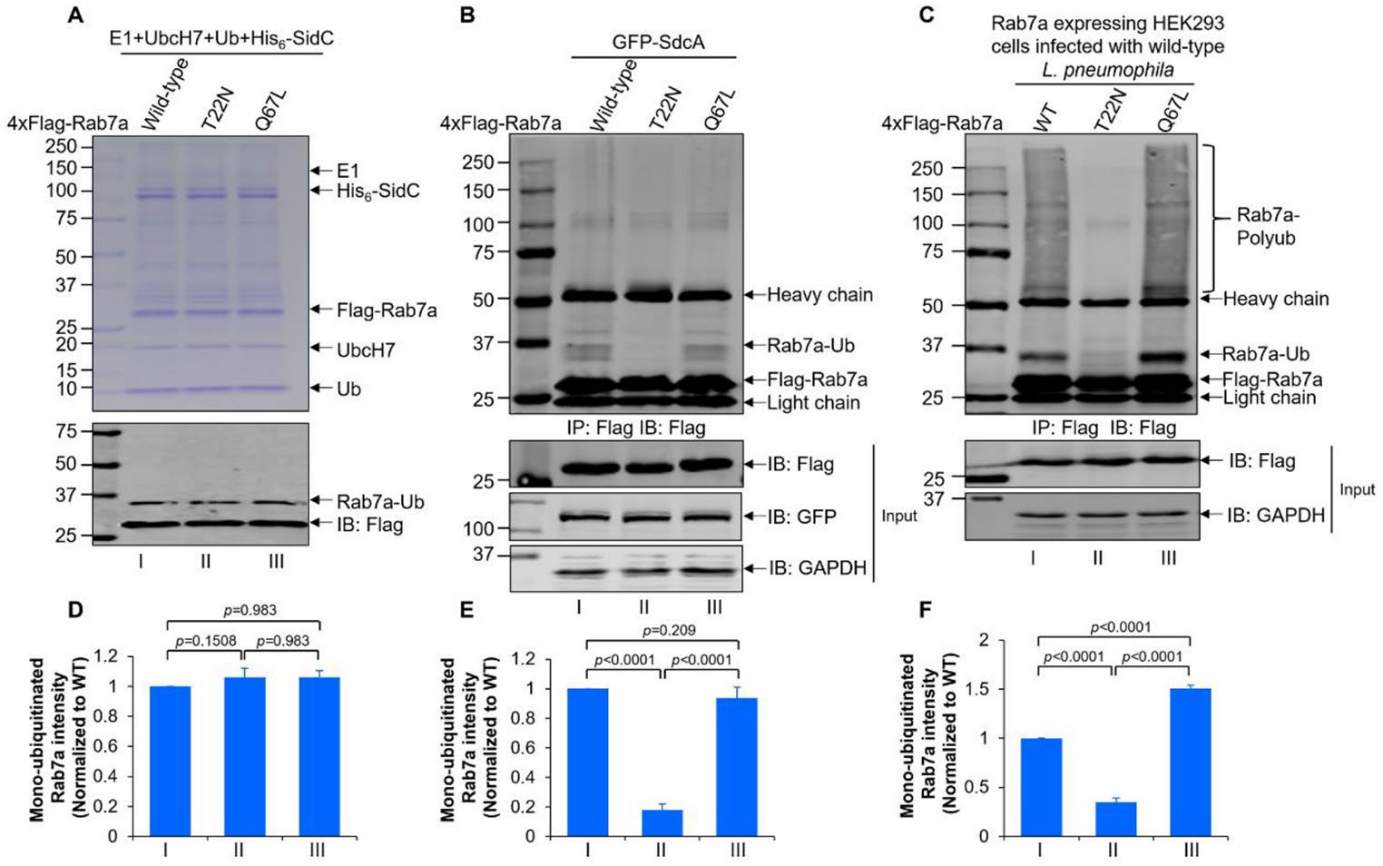
SidC/SdcA preferred to catalyze GTP-bound active Rab7 in vivo. (**A**) In vitro ubiquitination of dominant-positive (Q67L) and dominant-negative (T22N) Rab7 by SidC. Reactions containing E1, UbcH7, ubiquitin, SidC and wild-type Rab7 or T22N/Q67L mutated Rab7 were performed at 37 °C for 1 h. Proteins separated by SDS‒PAGE were visualized by Coomassie brilliant blue (CBB) staining or Western blot with the anti-Flag antibody. (**B**) 4xFlag-tagged Rab7, Rab7_T22N_, or Rab7_Q67L_ was coexpressed with GFP-SdcA in HEK293T cells. Cell lysates prepared 24 h post transfection were immunoprecipitated by anti-Flag beads. Cell lysates and the bead-enriched products were analyzed by Western blot using antibodies specific for Flag, GFP, or glyceraldehyde-3-phosphate dehydrogenase (GAPDH). (**C**) HEK293 cells transiently expressing FcγRII and 4xFlag-tagged Rab7, Rab7_T22N_, or Rab7_Q67L_ were further infected with wild-type *L. pneumophila* for 2 h at an MOI of 50. 4xFlag-tagged proteins were enriched by anti-Flag immunoprecipitation and detected by Western blot using Flag-specific antibodies. The anti-glyceraldehyde-3-phosphate dehydrogenase (GAPDH) blot was probed as the loading control. (**D-F**) Quantification of the mono-ubiquitinated Rab7a as shown in (A), (B), and (C) was determined by ImageJ. Panel D is related to panel A, panel E is related to panel B, and panel F is related to panel C. The results from panels A, B, and C are representative of three independent experiments. Values in panels D, E, and F are the mean ± SD of three independent tests

Rab GTPases (Rabs) cycle between a GDP-bound inactive form and a GTP-bound active form by the actions of a series of regulatory proteins, including guanine nucleotide exchange factors (GEFs) and GTPase-activating proteins (GAPs) [54]. Once activated, Rabs can interact with relevant downstream effectors to exert their regulatory roles [54]. To investigate whether Rab7 ubiquitination contributes to the avoidance of lysosomal maturation, we first evaluated the catalytic efficiency of SidC/SdcA for both dominant-negative (T22N) and dominant-positive (Q67L) mutants of Rab7. In the in vitro reactions, Rab7_T27N_ and Rab7_Q67L_ were equally ubiquitinated by SidC (Fig. 9A and D). However, when these Rab7 variants were transfected with GFP-SdcA in HEK239T cells, only wild-type Rab7 and Rab7_Q67L_ were modified by SdcA (Fig. 9B and E). Similarly, while wild-type Rab7 and Rab7_Q67L_ were evidently ubiquitinated in cells infected with wild-type *L. pneumophila*, ubiquitination of Rab7_T27N_ was barely detectable upon *L. pneumophila* infection (Fig. 9C and F). Hence, these data indicate that SidC catalyzed ubiquitination of Rab7 in the active state in vivo.

**Fig. 10:**
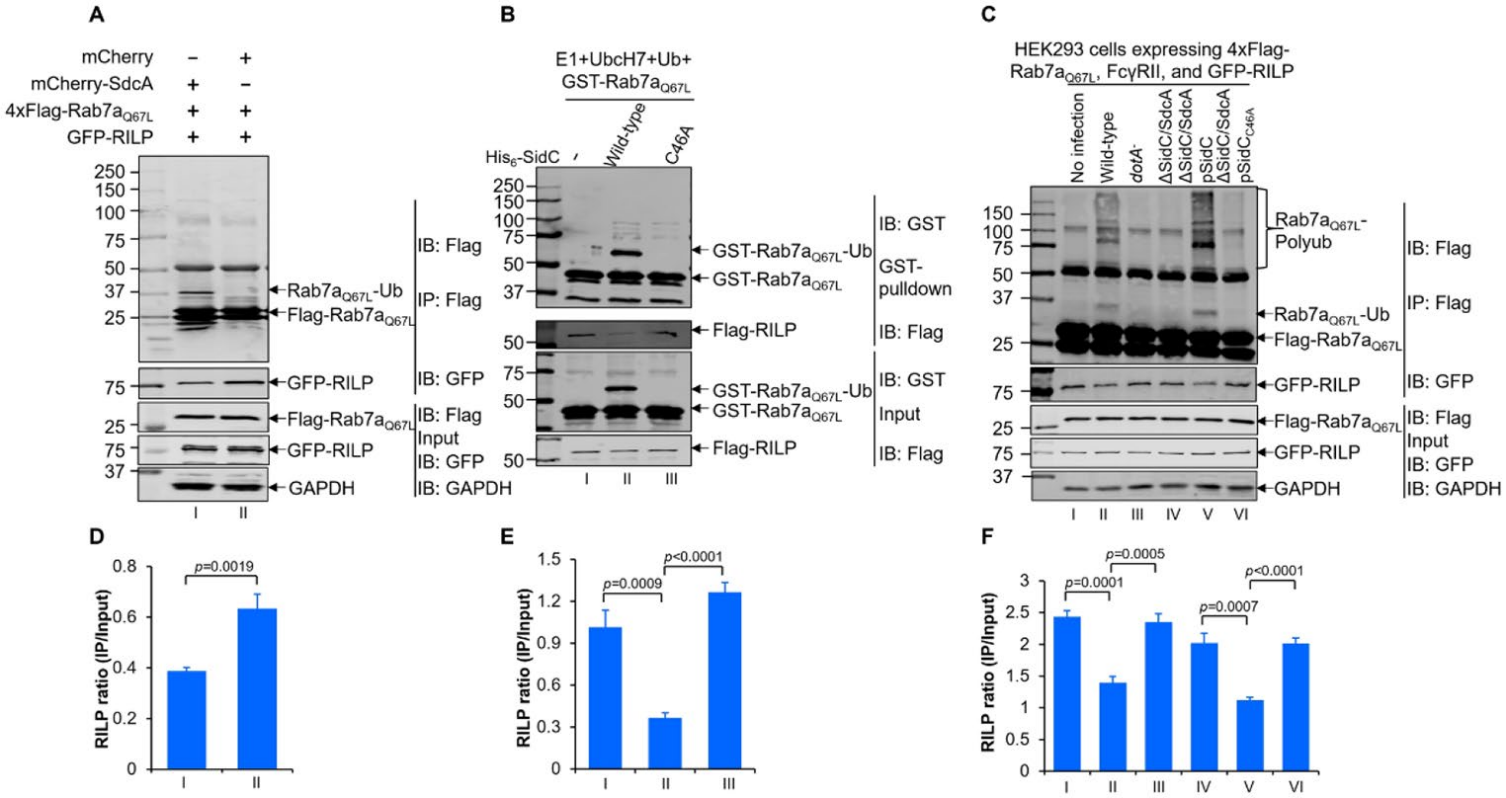
Ubiquitination of Rab7 decreases its interaction with the downstream effector Rab-interacting lysosomal protein (RILP). (**A**) HEK293T cells were transfected with 4xFlag-Rab7_Q67L_, GFP-RILP, and mCherry or mCherry-SdcA. Cells were lysed and immunoprecipitated with anti-Flag agarose. The cell lysates and the bead-associated proteins were detected by Western blot using anti-Flag and anti-GFP antibodies. Glyceraldehyde-3-phosphate dehydrogenase (GAPDH) was probed as the loading control. (**B**) In vitro ubiquitination reactions consisting of E1, UbcH7, Ub, His_6_-SidC/His_6_-SidC_C46A_, and GST-Rab7_Q67L_ were allowed to proceed for 2 h at 37 °C. Equal amount of recombinant His_6_-Flag-RILP was then added to each of the reaction and further incubated at 4 ^o^C for 1 h. After enrichment with the GST magnetic beads, the interaction between GST-Rab7_Q67L_ and His_6_-Flag-RILP was evaluated via Western blot using anti-GST or anti-Flag antibodies. (**C**) HEK293 cells transiently coexpressing 4xFlag-Rab7_Q67L_, FcγRII, and GFP-RILP were either uninfected (lane 2) or infected with the indicated *L. pneumophila* strains (lane 3–7) for 2 h (MOI = 50). After immunoprecipitation of the cell lysates by the anti-Flag beads, the association of RILP with Rab7_Q67L_ was assessed by Western blot analysis with Flag- or GFP-specific antibodies. Glyceraldehyde-3-phosphate dehydrogenase (GAPDH) was probed as the loading control. (**D-F**) The intensities of GFP-RILP were determined by ImageJ software. The GFP-RILP ratio was calculated by dividing the intensities of bead-bound RILP (IP) by those in the cell lysates (Input). Panels D, E, and F are related to panels A, B, and C, respectively. The data in panels A-C are representative of three independent experiments, while those in panels D-F are the mean ± SD of three independent assays

RILP is a downstream adaptor of Rab7 that is required for late endosome transport to lysosomes [55]. We attempted to examine whether Rab7 ubiquitination affects its ability to interact with downstream effectors. To this end, we first cotransfected 4xFlag-Rab7_Q67L_ and GFP-RILP with mCherry or mCherry-SdcA. After anti-Flag immunoprecipitation, the agarose-associated RILP was evaluated by Western blot analysis with anti-GFP antibodies. Strikingly, ubiquitination of Rab7_Q67L_ by SdcA in the transfected cells reduced its binding with RILP (Fig. 10A and D). Furthermore, we set up an in vitro reaction to generate ubiquitinated GST-Rab7_Q67L_ and used it for the pulldown assay. The data showed that, similar to the in vivo observations, ubiquitinated Rab7_Q67L_ bound significantly lower amounts of RILP than native Rab7_Q67L_ (Fig. 10B and E). Finally, we further infected 4xFlag-Rab7_Q67L_- and GFP-RILP-expressing HEK293 cells with relevant *L. pneumophila* strains. 4xFlag-Rab7_Q67L_ enriched by anti-Flag agarose was subjected to Western blot analysis with antibodies specific for GFP. Apparently, compared to uninfected and *dotA*^−^*L. pneumophila-*infected samples, the amount of Rab7_Q67L_-associated RILP was significantly decreased in cells receiving the wild-type strain (Fig. 10C and F). In line with SidC/SdcA-induced Rab7_Q67L_ ubiquitination, the interaction was not affected in Δ*sidC/sdcA L. pneumophila*-challenged cells. Importantly, introducing plasmids encoding wild-type SidC but not its E3-deficient mutant SidC_C46A_ into the mutant strain resulted in a similar decrease in Rab7/RILP interaction as the wild-type strain infected samples. Taken together, these data strongly indicate that Rab7 ubiquitination induced by SidC/SdcA impairs its interaction with the downstream effector RILP.

### Rab7 knockdown decreases *L. Pneumophila* intracellular replication

To investigate whether Rab7 is required for optimal *L. pneumophila* growth within host cells, we first used RNA interference (RNAi) to knockdown Rab7 expression in RAW264.7 cells (Figure S7A). Then, the cell lines were infected with the wild-type *L. pneumophila* strain, and intracellular replication was determined at 24, 48, and 72 h post infection. Importantly, at 48 h and 72 h post infection, there was a small but significant reduction in *L. pneumophila* replication within Rab7 knockdown cells compared to the growth in the control cells (Figure S7B). Therefore, Rab7-regulated vesicle trafficking pathways may be important for the construction of replication-permissive LCVs.

## Discussion

In recent decades, the biochemical characterization of *L. pneumophila* Dot/Icm effectors has revealed that at least 26 proteins are implicated in the regulation of host ubiquitin signaling [31]. However, the host substrates and the biological functions of most of these ubiquitination-modulating effectors remain poorly understood. SidC/SdcA were originally identified as tethering factors for ER-derived vesicles [43]. However, our structural and biochemical study revealed that the SidC N-terminal fragment possesses cysteine-based E3 ubiquitin ligase activity due to the presence of a Cys-His-Asp catalytic triad [35]. Importantly, this enzymatic activity is required for SidC/SdcA-mediated recruitment of ER-associated vesicles to bacterial vacuoles [35]. These observations contend that, instead of functioning as tethering factors, SidC/SdcA may ubiquitinate specific host proteins to interfere with vesicle trafficking pathways. Small GTPases are molecular switches and play critical roles in regulating intracellular vesicle transport [54]. Many *L. pneumophila* Dot/Icm substrates manipulate host vesicle trafficking by targeting specific small GTPases of the Rab [3], Arf [56], and Ran [57] families. In this study, ubiquitome analysis combined with biochemical validation allowed us to identify multiple Rab GTPases, including the previously reported Rab1 [44] and Rab10 [46], as ubiquitination substrates of SidC/SdcA. Importantly, in addition to Rabs (e.g., Rab1, Rab6, Rab8, and Rab10) that regulate secretory vesicle trafficking, Rabs involved in the control of endocytic trafficking, such as Rab5, Rab7, and Rab11, are also extensively ubiquitinated by SidC/SdcA during *L. pneumophila* infection. Therefore, SidC/SdcA could hijack diverse vesicle trafficking routes to promote the maturation of the bacterial phagosome. In a previous proteomic analysis of isolated LCVs, at least 14 small GTPases of the Rab family [41], in addition to Arf1 and Sar1, were associated with the phagosome membrane. Interestingly, 7 of these LCV-associated Rabs were confirmed to be ubiquitination substrates of SidC/SdcA. It has been shown that the association of Rab10 with LCVs is dependent on the E3 ligase activity of SidC/SdcA [46]. Our fluorescence microscopy investigation revealed that Rab7 is recruited to the bacterial vacuole in an identical way. Hence, SidC/SdcA-induced ubiquitination might represent a common strategy used by Rabs to localize to LCVs. This hypothesis is further supported by the results from a recently submitted manuscript by Adriana M. Steinbach et al. [58]. , where they showed that SidC/SdcA manipulates the association of Rab1 and Rab5 to the bacterial phagosomes. It was noted that some of the SidC/SdcA-targeted Rabs overlap with the phosphoribosyl-linked ubiquitination substrates of SidEs (e.g., Rab1 [13] and Rab6 [13]), raising the potential for cross-talk between conventional and noncanonical ubiquitination. Indeed, one recently submitted work demonstrated that multiple Rabs were cross-linked via unconventional polyubiquitin chains that were generated by SidEs and SidC/SdcA [59]. Although the biological consequences of cross-linked polyubiquitination on Rabs remain unclear, it was recently reported that the orchestrated actions of many *L. pneumophila* effectors including SidE and SidC families fine-tune the dynamics of Rab10 on LCVs [60].

SNAREs are the core components mediating membrane fusion [61]. The v-SNARE proteins on the vesicle membrane specifically pair with their cognate t-SNAREs, thereby driving the fusion of the vesicle membrane with the opposing membrane of the target organelle [61]. During the biogenesis of LCVs, the ER-associated v-SNARE Sec22b forms a noncanonical complex with plasma membrane (PM)-localized t-SNAREs (e.g., STX3 and STX4), allowing the fusion of ER-originated vesicles with PM-derived bacterial vacuoles [47]. Infection-stimulated ubiquitination of Sec22b appears to be important for the unconventional pairing event since deubiquitination of Sec22b by LotB could disassociate STX3 from Sec22b [48]. Indeed, our recent work revealed that the effector protein Lug15 is a novel E3 ligase mediating Sec22b ubiquitination, and this modification promotes the interaction between Sec22b and STX3 [49]. In addition to Rab small GTPases, in this study we also demonstrated that multiple t-SNARE proteins, including STX3, STX4, and STX7, were ubiquitinated by SidC/SdcA. Importantly, STX3/4 ubiquitination could improve their unconventional pairing with Sec22b. Collectively, SidC/SdcA could interfere with vesicle-mediated membrane trafficking at different stages.

Among the diverse small GTPases, Rab7 is a key regulator in the processes of endosomal membrane trafficking and phagosome-lysosome fusion [51, 62]. The active GTP-bound form of Rab7 interacts with the downstream effector RILP to induce the recruitment of dynein-dynactin motor complexes, thus controlling lysosomal transport [63]. Therefore, it is not surprising that Rab7 and its partners often serve as important targets in the pathogenesis of microorganisms, including fungi, viruses, and bacteria [64]. Despite the acquisition of Rab7, bacterial phagosomes containing *M. tuberculosis, M. bovis* BCG, *S. Typhimurium* or *L. pneumophila* avoid fusion with lysosomes [42, 65, 66]. Although the exact mechanism varies, disruption of the Rab7-RILP interaction represents a common strategy used by these bacterial pathogens to inhibit lysosomal fusion. For example, *M. bovis* BCG infection of macrophages impairs the interaction of Rab7 with RILP by an unknown mechanism, thereby contributing to the suppression of phagosomal maturation [63]. The *S. Typhimurium* type III secretion system effector SopD2 directly binds to Rab7 and inhibits its nucleotide exchange, which ultimately restricts the interaction of Rab7 with downstream effectors, including RILP [67]. Here, we illustrate a novel RILP exclusion mechanism via *L. pneumophila* effector SidC/SdcA-catalyzed ubiquitination of Rab7. Rab7 can undergo ubiquitination in cells by mammalian E3 ligases, including Parkin, primarily at the K38, K191, and K194 residues [50]. Importantly, a recent study reported that Rab7_K191/194R_ binds RILP more efficiently than the WT, suggesting a ubiquitination-dependent mechanism to regulate late endosome transport [68]. K194 is also a preferred ubiquitination site of Rab7 mediated by SidC/SdcA. Therefore, SidC/SdcA may, to some extent, mimic host E3 ligases to inhibit endosomal transport via disruption of the Rab7/RILP axis.

*L. pneumophila* is capable of modulating the function of host proteins through effectors possessing opposite biochemical activities, thereby imposing temporal and spatial regulation of host signaling pathways [4]. Our previous publication revealed the regulatory role of Lem27, an OTU family DUB protein of *L. pneumophila*, in Rab10 ubiquitination as well as its localization with the bacterial vacuoles induced by SidC/SdcA [69]. Whether Lem27 also works in concert with SidC/SdcA to regulate the dynamics of Rab7 on LCVs as well as the avoidance of lysosomal fusion in *L. pneumophila* infection requires further investigation.

In summary, our study demonstrates that *L. pneumophila* Dot/Icm substrates SidC/SdcA could ubiquitinate multiple Rab small GTPases and t-SANRE proteins during bacterial infection. SidC/SdcA-induced ubiquitination of STX3/STX4 promotes noncanonical SNARE paring with Sec22b, thus facilitating the fusion of ER-derived vesicles with phagosome membrane originated from the plasma membrane (Figure S8). Ubiquitination of Rab7 by SidC/SdcA disrupts its interaction with the downstream effector RILP, thereby blocking phagosomal fusion with the lysosomes (Figure S8). Our findings reveal the molecular mechanisms used by *L. pneumophila* to promote phagosomal maturation via hijacking host ubiquitin signaling.

## Materials and methods

### Bacterial strains, plasmids, cell lines, and growth media

The*L. pneumophila* strains used in this study were derivatives of the clinical isolate Philadelphia-1 [70]. *L. pneumophila* Δ*sidC/sdcA* and relevant complement strains were used in our previous study [35, 69]. *L. pneumophila* were grown on charcoal-N-(2-acetamido)-2-aminoethanesulfonic acid (ACES)–yeast extract (CYE) plates and in ACES-yeast extract (AYE) liquid medium at 37 °C. *E. coli* strains were cultivated in Luria broth (LB) media supplemented with kanamycin (30 µg/ml) or ampicillin (100 µg/ml) when needed. For the expression of recombinant proteins in *E. coli*, genes were either synthesized by Genescripts Corp. (Nanjing, China) or amplified from *L. pneumophila* genomic DNA. These genes were cloned and inserted into pET28a, pET-Sumo or pGEX6P-1 vectors for the expression of His_6_-, Sumo-, and GST-fusion proteins, respectively. For ectopic expression of proteins in mammalian cells, genes of interest were inserted into peGFPC1, pCDNA3.1-3xHA or pCMV-4xFlag vectors to generate GFP-, 3xHA-, and 4xFlag-tagged proteins. All the bacterial strains, plasmids, and primers used in the present study are listed in detail in Supplementary Table S1 and Table S2.

Murine macrophage-like Raw264.7 cells (ATCC) were cultured in RPMI 1640 (Sigma) medium in the presence of 10% heat-inactivated fetal bovine serum (FBS) (Biological Industries); HeLa, HEK293, and HEK293T cells were all obtained from ATCC and maintained in Dulbecco’s modified Eagle’s medium (DMEM) (Sigma) supplemented with 10% heat-inactivated FBS. All animal experiments were performed according to protocols approved by the Institutional Animal Care and Use Committee of Jilin University (NO: SY201902008). Bone marrow-derived macrophages (BMDMs) were collected from female A/J mice (6–8 weeks old) (Model Animal Research Center of Nanjing University, Nanjing, China) and differentiated for 7 days in bone marrow macrophage media containing 20% L929 cell supernatant-conditioned medium, 60% RPMI 1640, and 20% FBS. All cell types were grown at 37 °C with 5% CO_2_ in a humidified atmosphere.

### Protein expression and purification

The*E. coli* strain BL21 (DE3) was transformed with plasmids encoding the recombinant proteins. The bacteria were grown in LB media containing kanamycin or ampicillin at 37 °C with constant agitation to an OD_600nm_ of 0.6–0.8. Protein expression was then induced by adding IPTG (isopropyl β-D-1-thiogalactopyranoside) up to 200 µM. The cultures were further incubated at 18 °C for 14 h with constant shaking. The bacterial cells were then pelleted by centrifugation at 4000 x *g* for 20 min and resuspended in lysis buffer supplemented with protease inhibitor cocktail (Roche). The subsequent procedures were carried out at 4 °C. The suspensions were passed through a homogenizer (JN-mini, JNBIO, Guangzhou, China) three times at 1,100 psi to lyse the bacteria. Insoluble materials were removed from the bacterial lysates by centrifugation at 12,000 x *g* for 1 h. The supernatants were incubated with Ni-NTA beads (QIAGEN) or Glutathione Sepharose 4B GST-tagged protein purification resin (GE Healthcare) and incubated on an end-to-end rotator for 2 h. After extensive washing, the bead-bound proteins were eluted with 250 mM imidazole (for His_6_-fused proteins) or 20 mM reduced glutathione (for GST-tagged proteins). All purified recombinant proteins were dialyzed in buffer containing 300 mM NaCl, 20 mM Tris-HCl (pH 7.5), and 10% glycerol with Snakeskin dialysis tubing membranes. Proteins were stored at -80 °C prior to use in the biochemical reactions.

### Transfection and immunoprecipitation

For transfection, cells were seeded in culture plates and grown to a confluency of 80–90%. Plasmids were transfected with Lipofectamine 3000 transfection reagent (Invitrogen) according to the manufacturers’ instructions. After 24 h, the transfected cells were collected and lysed in RIPA buffer supplemented with 1x protease inhibitors at 4 °C for 10 min. Then, the lysates were centrifuged at 12,000 x *g* for 20 min at 4 °C to clear the cell debris. Anti-Flag agarose was added to the supernatants and incubated for an additional 4 h at 4 °C on an end-to-end rotator. The beads were washed with lysis buffer 3 times prior to the addition of 1x SDS sampling buffer.

### L. pneumophila infection

All*L. pneumophila* strains were cultured in AYE broth at 37 °C to the postexponential phase as judged by an OD_600nm_ of approximately 3.3–3.8. For infection of BMDMs, bacteria were directly added to the cells seeded on the plates (MOI = 0.05) for the indicated durations. For infection of FcγII-transfected HEK293 cells, *L. pneumophila* cultures were opsonized with rabbit anti-*L. pneumophila* antibodies diluted at 1:5000 for 30 min at 37 °C prior to infection. The opsonized bacteria were then used to infect transfected cells at MOIs of 50 for 2 h. The cell culture plates were centrifuged at 1000 x *g* for 5 min after adding the bacteria to facilitate contact between the bacteria and the cells. All infections were performed at 37 °C with 5% CO_2_ in a humidified atmosphere.

### Western blot analysis and immunostaining

For Western blot analysis, proteins separated by SDS‒PAGE were transferred to nitrocellulose membranes (Pall Life Sciences) using the wet transfer method. Following blocking with 5% w/v nonfat dry milk, the membrane was incubated with appropriate diluted primary antibodies in TBST buffer at room temperature (RT) with gentle shaking for 1 h. After washing 3 times with TBST buffer, the membranes were further probed with appropriate IRDye-conjugated secondary antibodies diluted at 1:10000 in TBST buffer for 1 h at RT. Then, the blot signals were detected by the Odyssey® CLx Infrared Imaging System (LI-COR Biosciences). The primary antibodies used in the present study are included in Table S2.

For immunostaining analysis, cells seeded on coverslips in 24-well plates were fixed with 4% paraformaldehyde in PBS at RT for 15 min. The cells were permeabilized with 0.2% Triton X-100 in PBS for 5 min, followed by blocking for 1 h with 4% goat serum in PBS. Then, the samples were probed with appropriate primary and secondary antibodies that were diluted in 4% goat serum. Nuclei were stained with DAPI for 5 min prior to mounting the coverslips on the glass slides. The antibodies used in the present study are shown in Table S2. The coverslips were subsequently visualized and imaged using a Zeiss LSM880 confocal microscope.

### In vitro protein ubiquitination assay

In vitro ubiquitination assays were set up to determine SidC/SdcA-catalyzed substrate ubiquitination. A 40 µl reaction mixture containing 50 mM Tris-HCl (pH 7.5), 0.2 µg E1, 1 µg UbcH7, 5 µg ubiquitin, 2 µg SidC/SdcA, 2 µg indicated substrate proteins, 1 mM DTT, 2 mM ATP, and 5 mM DTT was set up. All biochemical reactions were incubated at 37 °C for 1 h and quenched by the addition of 5x SDS sampling buffer. The formation of ubiquitinated species was analyzed by SDS‒PAGE followed by Coomassie brilliant blue (CBB) staining or Western blotting with specific antibodies. As indicated, reactions containing the catalytically inactive SidC/SdcA served as negative controls.

### GST pulldown assay

GST-Rab7 was mixed with E1, UbcH7, and ubiquitin in ubiquitination reaction buffer. Equal amounts of SidC or SidC_C46A_ were added to the mixture and incubated for 2 h to generate ubiquitinated Rab7. Then, the reaction was supplemented with equal amount of recombinant His_6_-Flag-RILP. After incubation at 4 °C for 1 h on an end-to-end rotator, 20 µl of prewashed glutathione sepharose resin was added and further incubated for 1 h. The beads were washed with cold PBS 3 times to remove unbound proteins and eluted with the addition of 1 x SDS sampling buffer. The association of GFP-RILP with Rab7 was evaluated by Western blotting with antibodies specific for Flag.

### Mass spectrometry analysis

The in vitro ubiquitination reactions containing E1, UbcH7, ubiquitin, Rab7, and SidC were resolved by SDS‒PAGE. Both the unmodified and modified Rab7 bands in the CBB-stained gel were excised and subjected to in-gel digestion by trypsin. The resulting Rab7 peptides were further analyzed by LC‒ MS/MS on a hybrid linear ion trap-Orbitrap mass spectrometer (Thermo Fisher Scientific). A 40-min LC gradient that ranged from 10 to 40% solvent B (100% ACN, 0.1% FA) was used.

### Replicative vacuole assay and growth curve

The replicative vacuole assay was carried out as previously described [44]. In brief, BMDMs seeded on coverslips in 24-well plates were challenged with relevant postexponential *L. pneumophila* strains at an MOI of 0.05 for 1 h. After removing untaken bacteria by washing the infection samples 3 times with warm PBS, infections were further proceeded for 7 h. Then, the samples were subjected to immunostaining with anti-*L. pneumophila* antibodies by the abovementioned procedures.

For the growth curve assay, BMDMs were seeded in 24-well plates and infected with *L. pneumophila* strains for 2 h at an MOI of 0.05. After clearing the extracellular bacteria, fresh culture media were added and further incubated for the indicated durations. Infected cells were lysed with saponin to a final concentration of 0.2% and incubated at 37 °C for 30 min. Then, the cell lysates were spotted onto CYE plates and grown in a 37 °C incubator for 4 days. The colony forming units (CFUs) were calculated to determine the intracellular replication of individual *L. pneumophila* strains.

### Knockdown of Rab7 using RNAi

The control-shRNA and Rab7-shRNA were designed by the Predesigned shRNA tool (Sigma‒Aldrich) and synthesized by Sangon Biotech (Shanghai, China). The annealed oligos were inserted into pLKO.1-eGFP-puro and cotransfected with pMD2. G and psPAX2 into HEK293T cells using Lipofectamine 3000. At 48 h post transfection, the lentiviral particle-containing supernatants were collected and added to RAW264.7 cell cultures. At 48 h post infection, the cells were cultured in medium containing 5 mg/mL puromycin for 7 days. Then, the virus was used to infect RAW264.7 cells using polybrene (10 mg/mL) for 5 days. The efficiency of Rab7 knockdown was evaluated by Western blotting using specific antibodies.

### Data analysis

For immunostaining, at least 100 LCVs were calculated for each sample. The protein levels of the Western blot images was assessed by ImageJ. Statistical analyses were performed using unpaired two-tailed Student’s *t* tests, and *p* < 0.05 was considered statistically significant.

### Electronic supplementary material

Below is the link to the electronic supplementary material.

Supplementary Material 1

## Data Availability

The datasets generated during the current study are available from the corresponding author on reasonable request.
