# Supplementary material for: Legionella effectors SidC/SdcA ubiquitinate multiple small GTPases and SNARE proteins to promote phagosomal maturation
Source: Cell Mol Life Sci. 2024 Jun 5;81(1):249. doi: 10.1007/s00018-024-05271-7 (PMC11335287; doi:10.1007/s00018-024-05271-7)
Supplement: Supplementary file 1 — Supplementary Material 1 [file 18_2024_5271_MOESM1_ESM.docx]

**Figure S1. Expression and translocation of SidC in the *L. pneumophila* strains. (A)** *L. pneumophila* strains were cultured in AYE broth to the postexponential phase (OD_600nm_=3.3-3.8). Equal amounts of bacteria were resuspended in 1x SDS sample buffer and boiled for 5 min. The expression of SidC in these strains was determined by Western blot analysis with a SidC-specific antibody. Anti-isocitrate dehydrogenase (ICDH) antibody was used to probe the membrane as the loading control. **(B)** Postexponential phase bacteria were used to infect RAW264.7 cells for 2 h at an MOI of 50. Infected cells were collected and lysed with 0.02% saponin. Cell lysates were probed with anti-SidC antibody to detect the translocation of SidC. The anti-glyceraldehyde-3-phosphate dehydrogenase (GAPDH) blot was used as the loading control. Data are representative of three independent experiments.

**
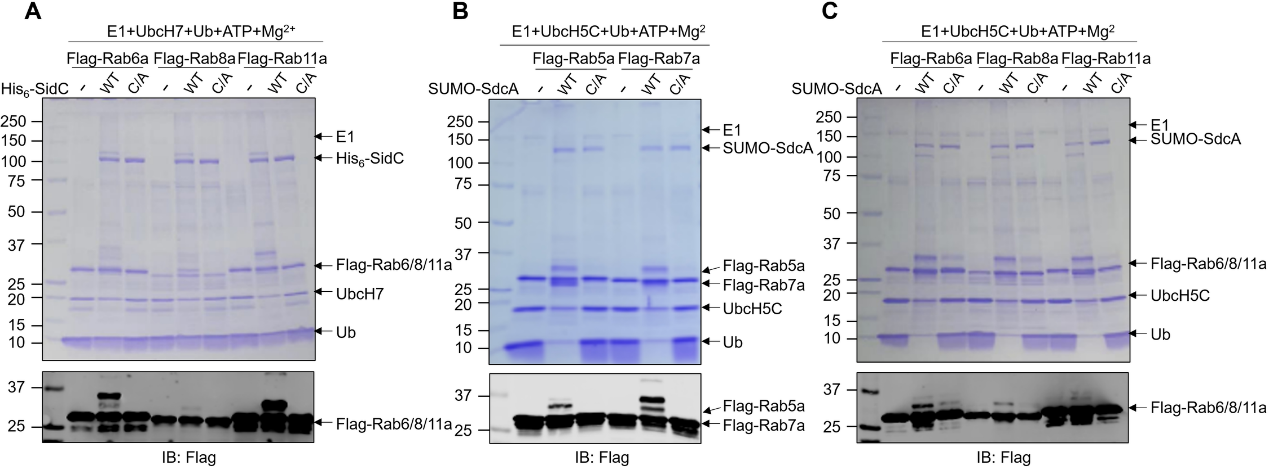
**

**Figure S2. *In vitro* ubiquitination of Rab small GTPase by SidC and SdcA. (A)** Ubiquitination of Rab6, Rab8, and Rab11 by SidC. The reaction mixtures containing E1, UbcH7, ubiquitin, and 4xFlag-Rab6/8/11 were incubated with His_6_-SidC or His_6_-SidC_C46A_ at 37 °C for 2 h. **(B-C)** Ubiquitination of Rab5, Rab6, Rab7, Rab8, and Rab11 by SdcA. Reactions containing E1, UbcH5C, ubiquitin, 4xFlag-Rab5/6/7/8/11, and Sumo-SdcA or Sumo-SdcA_C46A_ were allowed to proceed for 2 h at 37 °C. All ubiquitination reactions were terminated by the addition of 5x SDS sampling buffer and boiling for 5 min. Modification of these Rab proteins was observed by Coomassie brilliant blue (CBB) staining (upper) or Western blotting using Flag-specific antibodies (lower). The results are representative of three independent experiments.


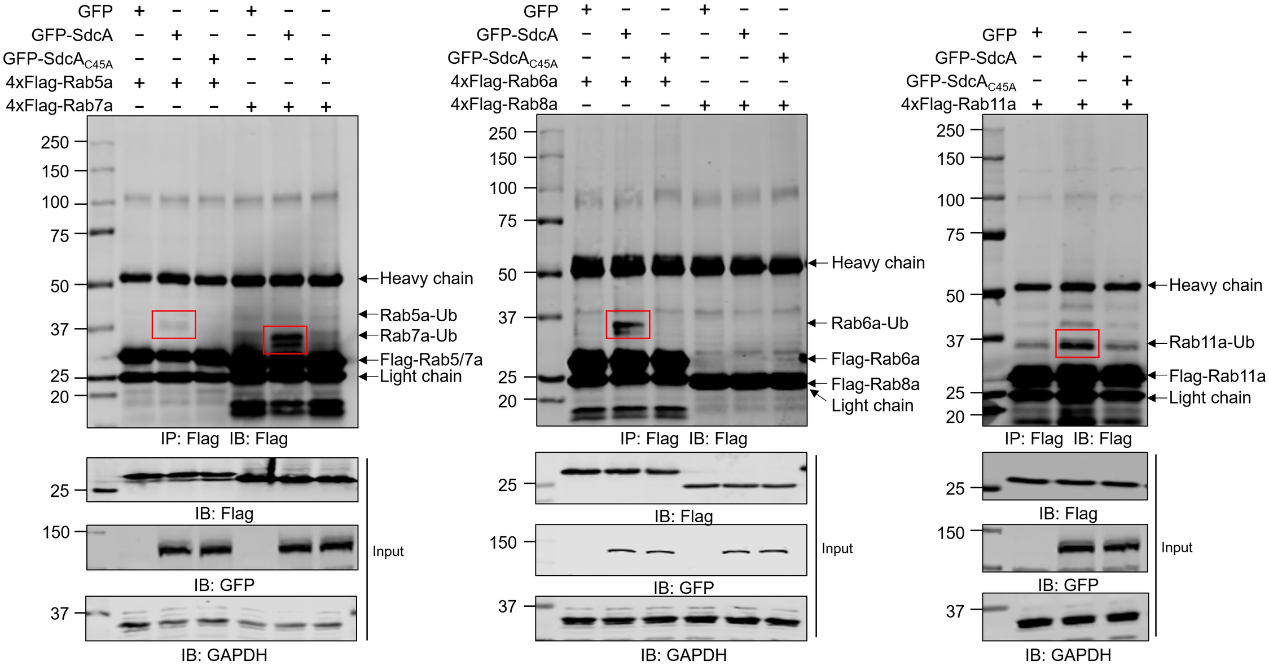


**Figure S3. Modification of Rab small GTPase by SdcA under transfection conditions.** Plasmids carrying each of the 4xFlag-tagged Rab proteins were cotransfected with GFP, GFP-SdcA, or GFP-SdcA_C45A_ into HEK293T cells. Immunoprecipitation of the cell lysates was performed using anti-Flag agarose. The bead-bound proteins, expression of the 4xFlag-tagged fusion proteins and the GFP-fusion constructs in the cell lysates were analyzed by Western blot with anti-Flag or anti-GFP antibodies. Glyceraldehyde-3-phosphate dehydrogenase (GAPDH) was probed as the loading control. Data are representative of three independent experiments.





**Figure S4. SidC/SdcA catalyzed the modification of Rab6, Rab8, and Rab11 in *L. pneumophila* infection.** (A-C) HEK293 cells were transfected to produce 4xFlag-Rab6 (A), 4xFlag-Rab8 (B), and 4xFlag-Rab11 for 24 h. Cells were uninfected or challenged with relevant *L. pneumophila* strains for 2 h prior to cell lysis. 4xFlag-tagged proteins were enriched by anti-Flag immunoprecipitation of the cell lysates and detected by Western blot analysis with anti-Flag antibodies. Cell lysates were probed with anti-Flag and anti-glyceraldehyde-3-phosphate dehydrogenase (GAPDH) antibodies to show the expression of 4xFlag fused proteins as well as equal loading of the samples. Data are representative of three independent experiments.


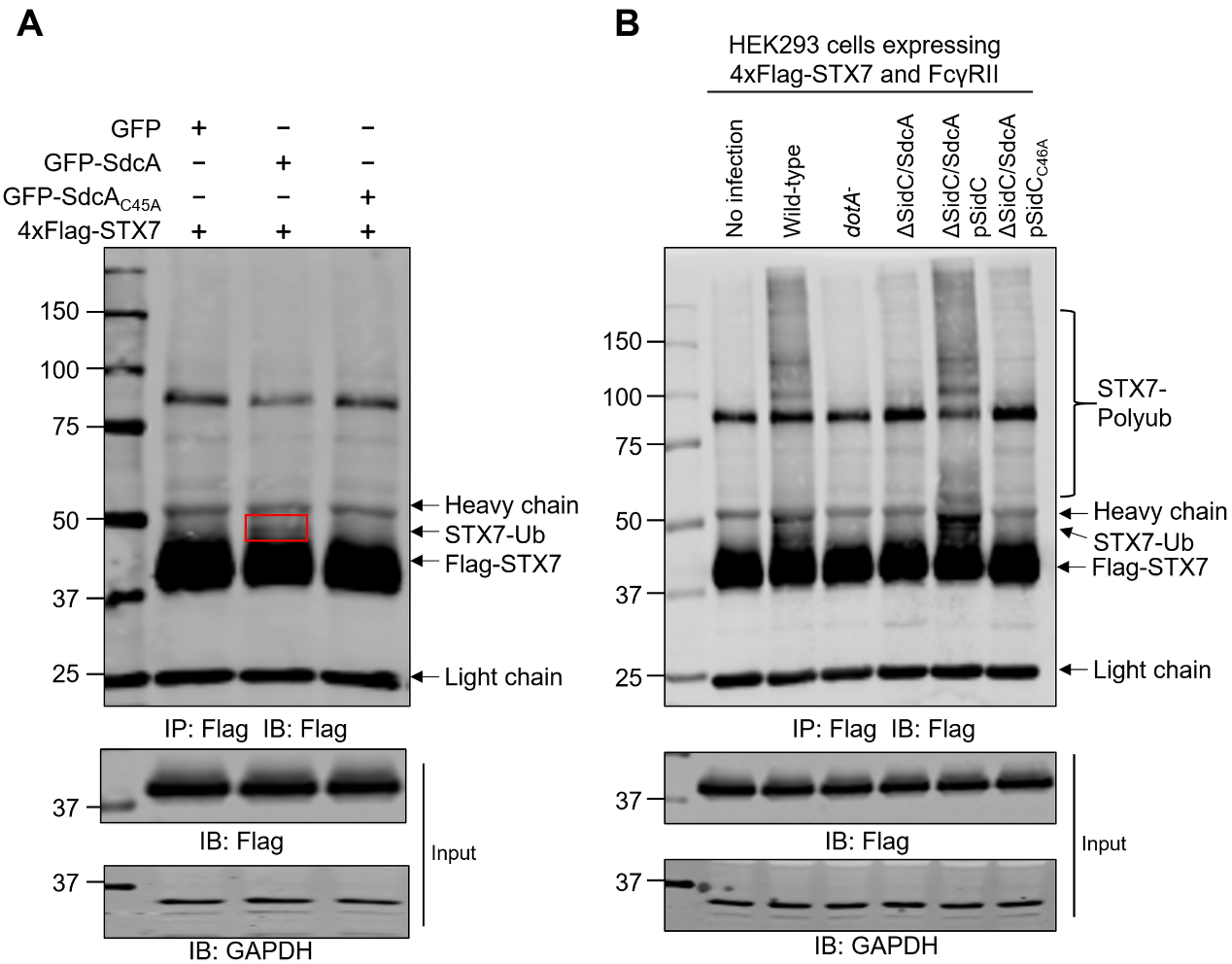


**Figure S5. SidC/SdcA-induced modification of the t-SNARE protein syntaxin 7 (STX7) in transfected and infected cells. (A)** 4xFlag-STX7 was coexpressed with GFP, GFP-SdcA, or GFP-SdcA_C45A_ in HEK293T cells. 4xFlag-STX7 enriched from the cell lysates by anti-Flag immunoprecipitation was further detected by Western blot using an antibody recognizing Flag. **(B)** 4xFlag-STX7-producing HEK293 cells were either uninfected or infected with the indicated *L. pneumophila* strains for 2 h (MOI=50). After immunoprecipitation of the cell lysates with anti-Flag beads, the enriched 4xFlag-STX7 was visualized by Western blot with the anti-Flag antibody. The cell lysates were also probed with anti-Flag and anti-glyceraldehyde-3-phosphate dehydrogenase (GAPDH) antibodies to indicate 4xFlag-STX7 expression and equal loading of the samples, respectively. The data shown in panels A and B are representative of three independent experiments.

**
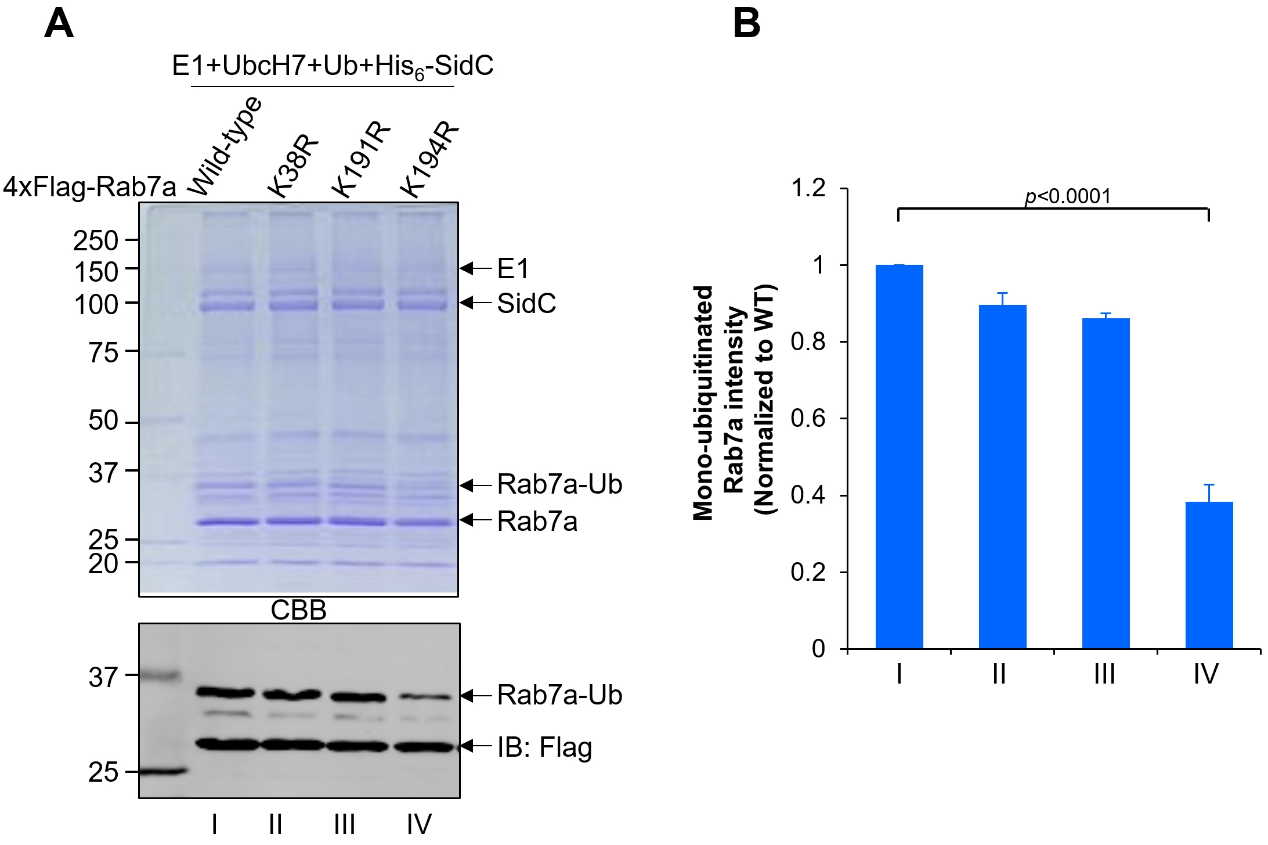
Figure S6. *In vitro* ubiquitination of Rab7_K38R_, Rab7_K191R_, and Rab7_K194R_ by SidC. (A)** The mixtures containing E1, UbcH7, ubiquitin, His_6_-SidC and each of the Rab7 lysine mutants were reacted at 37 °C for 30 min. Reactions were stopped by the addition of 5x SDS sample buffer and separated by SDS‒PAGE. Ubiquitination of Rab7 and its derivatives was detected by Coomassie brilliant blue (CBB) staining or Western blotting with an anti-Flag antibody. **(B)** Quantification of the mono-ubiquitinated Rab7a as shown in (A) was determined by ImageJ. Panel A is one representative of three independent experiments. Values in panels B are the mean ± SD of three independent tests.


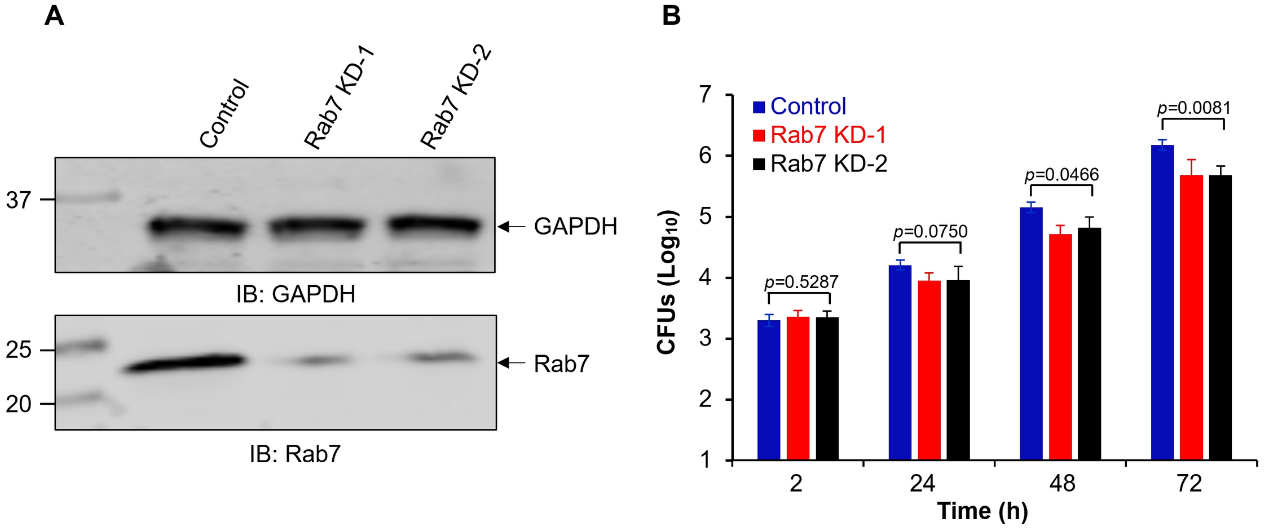


**Figure S7. Knockdown of Rab7 in Raw264.7 cells decreases intracellular *L. pneumophila* replication. (A)** Validation of Rab7 knockdown (KD). Cell lysates resolved by SDS‒PAGE were analyzed by Western blotting with an anti-Rab7 antibody. The anti-glyceraldehyde-3-phosphate dehydrogenase (GAPDH) antibody was used to probe the membrane as a loading control. **(B)** Intracellular proliferation of wild-type *L. pneumophila* within the control and Rab7 knockdown cells. Cells were challenged with postexponential phase bacteria at an MOI of 0.05. At 2, 24, 48, and 72 h post infection, infected cells were lysed with 0.02% saponin and plated on CYE agar plates. The bacterial numbers were counted after incubation at 37 °C for 4-5 days. The results in panel B are one representative of three independent experiments performed in triplicate.

**
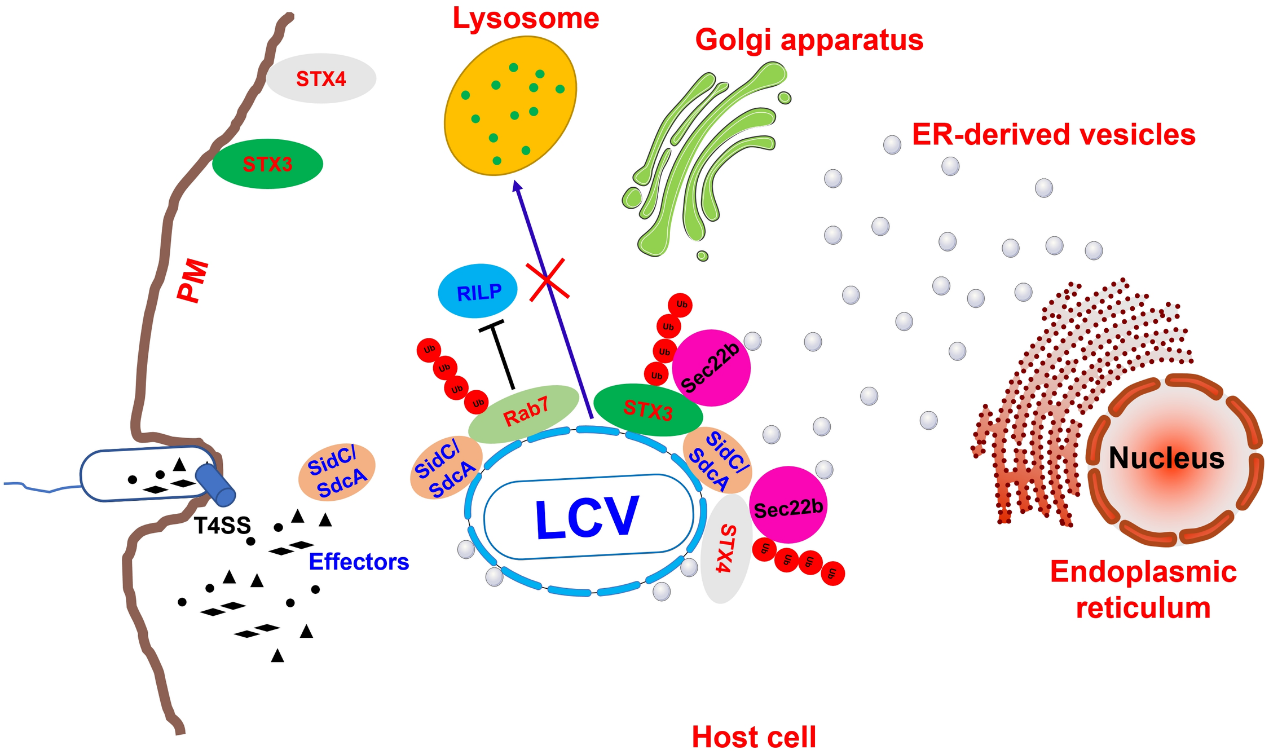
Figure S8. *L. pneumophila* effectors SidC/SdcA promotes phagosomal maturation during infection.** Upon translocation into the host cells via the Dot/Icm secretion system, SidC/SdcA ubiquitinate multiple Rab small GTPases and t-SNARE proteins. Ubiquitination of syntaxin 3 (STX3) and syntaxin 4 (STX4) by SidC/SdcA increases their noncanonical pairing with v-SNARE protein Sec22b, promoting fusion of ER-derived vesicles with the phagosome membranes. SidC/SdcA-catalyzed ubiquitination of Rab7 suppresses its binding with Rab-interacting lysosomal protein (RILP), thus allowing avoidance of phagosome-lysosome fusion.

**Table S1. Primers used in this study**

| **Primers** | **Sequences** | **Usage** |
| --- | --- | --- |
| *rab5a*-BamHI-F | ctgggatccatggctagtcgaggcg | To clone *rab5a* fragment |
| *rab5a* -SalI-R | ctggtcgacttagttactacaacactg | To clone *rab5a* fragment |
| *rab6a* -BamHI-F | ctgggatccatgtccacgggcggag | To clone *rab6a* fragment |
| *rab6a* -SalI-R | ctggtcgacttagcaggaacagcct | To clone *rab6a* fragment |
| *rab7a* -BamHI-F | ctgggatccatgacctctaggaaga | To clone *rab7a* fragment |
| *rab7a* -SalI-R | ctggtcgactcagcaactgcagctt | To clone *rab7a* fragment |
| *rab8a*-BamHI-F | ctgggatccatggcgaagacctacg | To clone *rab8a* fragment |
| *rab8a* -SalI-R | ctggtcgactcacagaagaacacat | To clone *rab8a* fragment |
| *rab11a*-BamHI-F | ctgggatccatgggcacccgcgacg | To clone *rab11a* fragment |
| *rab11a*-SalI-R | ctggtcgacttagatgttctgacag | To clone *rab11a* fragment |
| *syntaxin3*-BamHI-F | ctgggatccatgaaggaccgtctgg | To clone *syntaxin3* fragment |
| *syntaxin3*-SalI-R | ctggtcgacttaattcagcccaacg | To clone *syntaxin3* fragment |
| *syntaxin4*-BamHI-F | ctgggatccatgcgcgacaggaccc | To clone *syntaxin4* fragment |
| *syntaxin4*-SalI-R | ctggtcgacttatccaaccactgtg | To clone *syntaxin4* fragment |
| *syntaxin7*-BamHI-F | ctgggatccatgtcttacactccaggag | To clone *syntaxin7* fragment |
| *syntaxin7*-SalI-R | ctggtcgactcagtggttcaatcccca | To clone *syntaxin7* fragment |
| *RILP*-BglII-F | ctgagatctatggagcccaggaggg | To clone *RILP* fragment |
| *RILP*-SalI-R | ctggtcgactcaggcctctggggcg | To clone *RILP* fragment |
| *ub*-BamHI-F | ctgggatccatgcagattttcgtga | To clone *ub* fragment |
| *ub*-SalI-R | ctggtcgacttaaccaccacgaagt | To clone *ub* fragment |
| *sec22b*-BamHI-F | ctgggatccatggtgttgctaacaa | To clone *sec22b* fragment |
| *sec22b*-SalI-R | ctggtcgactcacagccaccagaat | To clone *sec22b* fragment |
| *sdcA*-BamHI-F | ctgggatccgtgatgaacatggttg | To clone *sdcA* fragment |
| *sdcA*-SalI-R | ctggtcgacctatattgtattcctaacag | To clone *sdcA* fragment |
| *sidC*-BamHI-F | ctgggatccatggtgataaacatgg | To clone *sidC* fragment |
| *sidC*-SalI-R | ctggtcgacctatttctttataattcccg | To clone *sidC* fragment |
| *sidC*_C46A_-F | gataataccgctcaaacagca | For mutagenesis |
| *sidC*_C46A_-R | tgctgtttgagcggtattatc | For mutagenesis |
| *sdcA*_C45A-_-F | gataataccgctgaaacaaca | For mutagenesis |
| *sdcA*_C45A_-R | tgttgtttcagcggtattatc | For mutagenesis |
| *rab7a*_T22N_-F | gtcgggaagaattcactcatg | For mutagenesis |
| *rab7a*_T22N_-R | catgagtgaattcttcccgac | For mutagenesis |
| *rab7a*_Q67L_-F | acagcaggactggaacggttc | For mutagenesis |
| *rab7a*_Q67L_-R | gaaccgttccagtcctgctgt | For mutagenesis |
| *rab7a*_K38R_-F | aatcagtacagagccacaata | For mutagenesis |
| *rab7a*_K38R_-R | tattgtggctctgtactgatt | For mutagenesis |
| *rab7a*_K191R_-F | gaacctatcagactggacaag | For mutagenesis |
| *rab7a*_K191R_-R | cttgtccagtctgataggttc | For mutagenesis |
| *rab7a*_K194R_-F | aaactggacaggaatgaccgg | For mutagenesis |
| *rab7a*_K194R_-R | ccggtcattcctgtccagttt | For mutagenesis |
| *rab7a*_K6R_-F | acctctaggaggagagtgttgctg | For mutagenesis |
| *rab7a*_K6R_-R | cagcaacactctcctcctagaggt | For mutagenesis |
| *rab7a*_K32R_-F | gtactgattgctgaatctcctattcacatactggttcatg | For mutagenesis |
| *rab7a*_K32R_-R | catgaaccagtatgtgaataggagattcagcaatcagtac | For mutagenesis |
| non-targeting randomized sequence | ccggatctcgcttgggcgagagtaagttcaagagacttactctcgcccaagcgagatttttttggtacc | Control of *rab7a* knockdown cells |
| non-targeting randomized sequence | aattggtaccaaaaaaatctcgcttgggcgagagtaagtctcttgaacttactctcgcccaagcgagat | Control of *rab7a* knockdown cells |
| *rab7a*-Knockdown  -F-1 | ccggtgctgtgttctggtgtttgatttcaagagaatcaaacaccagaacacagcattttttggtacc | To construct *rab7a* knockdown cells |
| *rab7a*-Knockdown  -R-1 | aattggtaccaaaaaatgctgtgttctggtgtttgattctcttgaaatcaaacaccagaacacagca | To construct *rab7a* knockdown cells |
| *rab7a*-Knockdown  -F-2 | ccgggcggcagtattctgtacagtattcaagagatactgtacagaatactgccgcttttttggtacc | To construct *rab7a* knockdown cells |
| *rab7a*-Knockdown  -R-2 | aattggtaccaaaaaagcggcagtattctgtacagtatctcttgaatactgtacagaatactgccgc | To construct *rab7a* knockdown cells |

**Abbreviations:** RILP, Rab-interacting lysosomal protein; Ub, Ubiquitin

**Table S2. Resources of key materials used for this study**

| **Reagent or Resource** | **Source** | **Identifier** |
| --- | --- | --- |
| **Antibodies** |  |  |
| Rabbit anti-GFP | Sigma | catalog # AB10145 |
| Rabbit anti-ICDH | Sigma | catalog # ABS2090 |
| Rabbit anti-Rab7 | Cell Signaling | catalog # 9367 |
| Rabbit anti-GAPDH | Abcam | catalog # ab9485 |
| Rabbit anti-LAMP1 | Abcam | catalog # ab278043 |
| Rabbit anti-*Legionella* | Thermo | catalog # PA1-7227 |
| Mouse anti-FLAG | Sigma | catalog # F1804 |
| Mouse anti-Ub | Santa Cruz | catalog # sc-8017 |
| **Bacterial strains** |  |  |
| *Escherichia coli* DH5α | Tangen | N/A |
| *Escherichia coli* BL21/DE3 | Tangen | F^-^ *omp*T *hsdS*B (rB^-^ mB^-^) *gal* *dcm* (DE3) |
| *Legionella pneumophila* Philadelphia-1 strain Lp02 | (Berge & Isberg, 1993) | Philadelphia-1 *rpsL hsdR thyA* |
| *L. pneumophila* Lp03 | (Berge & Isberg, 1993) | Lp02 *dotA*^-^ |
| Lp02 *∆sidC/sdcA* | (Hsu F & Mao, 2014) | Lp02*∆sidC∆sdcA* |
| *∆sidC/sdcA* (pZL507) | (Liu et al., 2020) | Lp02*∆sidC∆sdcA* + pZL507 |
| *∆sidC/sdcA*(psidC) | (Liu et al., 2020) | Lp02*∆sidC∆sdcA* + pZL507-SidC |
| *∆sidC/sdcA*(psidC_C46A_) | (Liu et al., 2020) | Lp02*∆sidC∆sdcA* + pZL507-SidC_C46A_ |
| **Constructs** |  |  |
| pET28a | Novagen | Kan^R^, *E. Coli* expression vectors for His-tagged proteins |
| pET28a-Flag | This study | Flag tag before pET28a MCS |
| pET28a-Flag-Rab5a | This study | N/A |
| pET28a-Flag-Rab6a | This study | N/A |
| pET28a-Flag-Rab7a | This study | N/A |
| pET28a-Flag-Rab8a | This study | N/A |
| pET28a-Flag-Rab11a | This study | N/A |
| pET28a-Flag-RILP | This study | N/A |
| pGEX-6P-1 | GE | Amp^R^, *E. Coli* expression vectors for GST-tagged proteins |
| pGEX-6P-1-Rab7a | This study | N/A |
| pGEX-6P-1-Rab7a_Q67L_ | This study | N/A |
| pQE30 | Novagen | Amp^R^, *E. Coli* expression vectors for His-tagged proteins |
| pQE30-SidC | This study | N/A |
| pQE30-SidC_C46A_ | This study | N/A |
| pETSUMO | Novagen | Kan^R^, *E. Coli* expression vectors for His-tagged proteins |
| pETSUMO-SdcA | This study | N/A |
| pETSUMO-SdcA_C45A_ | This study | N/A |
| pZL507 | This study | For expression His_6_-tagged protein in *L. pneumophila* |
| pZL507-SidC | This study | N/A |
| pZL507-SidC_C46A_ | This study | N/A |
| peGFPC1 | Clontech | For expressing N-terminal GFP fusion proteins in mammalian cells |
| peGFPC1-SdcA | This study | N/A |
| peGFPC1-SdcA_C45A_ | This study | N/A |
| peGFPC1-Sec22b | This study | N/A |
| pCMV4×Flag | This study | For expressing N-terminal 4×Flag fusion proteins in mammalian cells |
| pCMV4×Flag-Rab5a | This study | N/A |
| pCMV4×Flag-Rab6a | This study | N/A |
| pCMV4×Flag-Rab7a | This study | N/A |
| pCMV4×Flag-Rab8a | This study | N/A |
| pCMV4×Flag-Rab11a | This study | N/A |
| pCMV4×Flag-syntaxin3 | This study | N/A |
| pCMV4×Flag-syntaxin4 | This study | N/A |
| pCMV4×Flag-syntaxin7 | This study | N/A |
| pCMV4×Flag-Rab7a_T22N_ | This study | N/A |
| pCMV4×Flag-Rab7a_Q67L_ | This study | N/A |
| pCMV4×Flag-Rab7A_K38R_ | This study | N/A |
| pCMV4×Flag-Rab7A_K191R_ | This study | N/A |
| pCMV4×Flag-Rab7A_K194R_ | This study | N/A |
| pCMV4×Flag-Rab7A_K6R_ | This study | N/A |
| pCMV4×Flag-Rab7A_K32R_ | This study | N/A |

**Abbreviations:** RILP, Rab-interacting lysosomal protein; Ub, Ubiquitin; ICDH, Isocitrate dehydrogenase; GAPDH, glyceraldehyde-3-phosphate dehydrogenase; LAMP1, Lysosomal associated membrane protein 1.
